# An Approach to Linguistic Multiple Attribute Decision-Making Based on Unbalanced Linguistic Generalized Heronian Mean Aggregation Operator

**DOI:** 10.1155/2018/1404067

**Published:** 2018-06-12

**Authors:** Bing Han, Huayou Chen, Jiaming Zhu, Jinpei Liu

**Affiliations:** ^1^School of Mathematical Sciences, Anhui University, Hefei, Anhui 230601, China; ^2^School of Business, Anhui University, Hefei, Anhui 230601, China; ^3^Edward P. Fitts Department of Industrial and Systems Engineering, North Carolina State University, Raleigh, NC 27695, USA

## Abstract

This paper proposes an approach to linguistic multiple attribute decision-making problems with interactive unbalanced linguistic assessment information by unbalanced linguistic generalized Heronian mean aggregation operators. First, some generalized Heronian mean aggregation operators with unbalanced linguistic information are proposed, involving the unbalanced linguistic generalized arithmetic Heronian mean operator and the unbalanced linguistic generalized geometric Heronian mean operator. For the situation that the input arguments have different degrees of importance, the unbalanced linguistic generalized weighted arithmetic Heronian mean operator and the unbalanced linguistic generalized weighted geometric Heronian mean operator are developed. Then we investigate their properties and some particular cases. Finally, the effectiveness and universality of the developed approach are illustrated by a low-carbon tourist instance and comparison analysis. A sensitivity analysis is performed as well to test the robustness of proposed methods.

## 1. Introduction

As an important part of multicriteria decision-making, multiple attribute decision-making (MADM) [1] and multiobjective decision-making build up the multicriteria decision-making system. The MADM concentrates research on discrete finite alternatives. The essence of MADM is ranking for the given alternatives and selecting the most desirable one. In order to integrate the individual preference value into a collective one, various operators have been presented during the past few years, such as the ordered weighted average (OWA) operator [2] which pays attention to the ordered position of each input datum, the ordered weighted geometric (OWG) operator [3], the dependent uncertain OWA (DUOWA) operator [4, 5], and the generalized OWA (GOWA) operator by adding an attitude parameter [6]. Zhou and Chen [7] investigated the continuous generalized OWA operator. Merigo [8, 9] presented the induced uncertain heavy OWA operators and induced generalized OWA (IGOWA) operator by using induced variables. Liao and Xu [10, 11] investigated the hybrid aggregation operators which consider the weight of arguments and their positions simultaneously. Liu et al. [12] presented some q-Rung Orthopair Fuzzy Aggregation Operators which could describe the space of uncertain information broadly.

However, the above aggregation operators have one thing in common: all input arguments are irrelevant, which is not realistic. The Heronian mean (HM) operator can overcome the drawback and has been improved to be an aggregation operator in [13]. Subsequently, a new range of extensions have been proposed, like the generalized Heronian mean (GHM) operator [14, 15], the intuitionistic fuzzy geometric HM (IFGHM) operator [16], the uncertain linguistic Heronian mean (ULHM) aggregation operators [17, 18], partitioned Heronian means operator [19], and Heronian aggregation operators of intuitionistic fuzzy numbers [20]. The Heronian mean operator has some particular characteristics that the others do not have. Contrasting the Choquet integral or power average operator which stresses on weight changes subjectively or objectively, Heronian mean focuses on aggregated arguments themselves. For a set of criteria values *e*_*i*_(*i* = 1, ⋯, *n*), the Bonferroni mean operator only considers the correlation between *e*_*i*_ and *e*_*j*_(*i* ≠ *j*). However, the relationship between *e*_*i*_ and itself can not be considered. The Heronian mean operator can solve the correlation of both the different criteria values *e*_*i*_, *e*_*j*_(*i* < *j*) and the criteria value *e*_*i*_ itself.

With the development of society, the decision-making information is more and more fuzzy or uncertain [21, 22]. It is more suitable and reasonable to express the preference in the form of linguistic information rather than real number. Some fuzzy linguistic approaches were firstly introduced by Zadeh [23]. Later on, a series of extended linguistic term sets have been developed, such as intuitionistic linguistic term set (ILTS) [24–28], 2-tuple linguistic term set (2TLTS) [19, 29–33], virtual linguistic term set (VLTS) [34], probabilistic linguistic term set (PLTS) [35], and hesitant fuzzy linguistic tern set (HFLTS) [36]. The ILTS was introduced by Wang and Li [36] which has three main parts: linguistic terms, membership function, and nonmembership function. Herrera and Martinez [37] presented 2-tuple linguistic (2TL) model which can avoid information loss validly. To preserve all the given information, Xu [34] extended the original linguistic term set to a continuous linguistic term set and introduced the concept of the virtual linguistic term. Some researchers have reported that the computational models of both the 2-tuple linguistic model and the virtual linguistic model are equivalent [38, 39]. In consideration of the possible uncertainties in linguistic expression, the probability linguistic term set (PLTS) [35] was developed through adding the probabilities without loss of any original linguistic variables. The HFLTS, combining the LTS and the HFS, was introduced by Rodríguez et al. [40]. It is a more reasonable information expression form, which can be used to describe people's subjective cognitions.

Obviously, the above linguistic aggregation operators are based upon symmetrically and uniformly placed linguistic term set. However, it is necessary to give evaluations by using nonsymmetrically and nonuniformly distributed linguistic terms [41] in some cases. For example, when assessing a person's ability, the linguistic term set used by experts is {extremely bad, bad, medium, almost good, good, quite good, very good, extremely good, perfect}. The number of the terms lying on the left of the central term “medium” (two) is less than that on the right one (six). To overcome the drawback, the unbalanced linguistic representation model has been presented in [42]. Subsequently, the unbalanced linguistic aggregation operators were introduced, for instance, the unbalanced linguistic OWG (ULOWG) operator [43], the unbalanced linguistic weighted OWA (ULWOWA) operator [41], and the unbalanced linguistic power average (ULPA) operator [44]. Furthermore, unbalanced linguistic aggregation operators in risk analysis were also investigated in [45, 46].

Through the above analysis, it is very important and necessary to develop the Heronian mean operator to cope with unbalanced linguistic information. Thus, the aim of this paper is to solve multiple attribute decision-making problems in which the evaluation information is correlative unbalanced linguistic information by combining the Heronian mean operator with unbalanced linguistic variables. We first introduce the unbalanced linguistic generalized arithmetic Heronian mean (ULGAHM) operator and the unbalanced linguistic generalized geometric Heronian mean (ULGGHM) operator. The most crucial advantage of these operators is that they could take into account correlation of input variables and deal with unbalanced linguistic information. For the situation that different attributes have different degrees of importance, the unbalanced linguistic generalized weighted arithmetic Heronian mean (ULGWAHM) operator and the unbalanced linguistic generalized weighted geometric Heronian mean (ULGWGHM) operator are presented and applied to MADM problems. The motivation of this paper is reposed on the following facts:

(i) The existing aggregation operators with unbalanced linguistic information are mainly concentrated on the OWA and OWG operator. There was less research about Heronian mean operator with unbalanced linguistic information.

(ii) The generalized Heronian mean aggregation operators can reflect the relationship of both the different criteria values *e*_*i*_, *e*_*j*_(*i* < *j*) and the criteria value *e*_*i*_ itself. In addition, they have flexible parameters *p* and *q*, and we could select the appropriate *p* and *q* to meet the different actual requirements.

(iii) Zou [43] just considered the weights of criteria in unbalanced linguistic environment. Meng [44] considered the weights of both experts and attributes. However, both of them ignore the relationship of input arguments. The multiple attribute decision-making [43, 44] can not deal with the situation where the assessment is in form of interrelated unbalanced linguistic information. Jiang [45] emphasized the changing of the weight of aggregation operator not the input arguments themselves. These new Heronian mean operators with unbalanced linguistic information could be used to solve above cases effectively.

The rest of the paper is arranged as follows: Section 2 introduces some basic concepts and notions. Some operational laws for unbalanced linguistic 2-tuple are defined in Section 3. In Section 4, some existing Heronian mean operators are reviewed and further we developed some new unbalanced linguistic generalized Heronian mean operators and investigated the properties and particular cases.  Section 5 presents the multiple attribute decision-making problem with unbalanced linguistic information. Subsequently, an actual example is given in Section 6.  Section 7 concludes the comparison analyses with other methods. Finally, the paper is summarized in Section 8.

## 2. Preliminaries

In this section, we briefly review the linguistic approach and the unbalanced linguistic representation model.

### 2.1. The Linguistic Approach

As an approximate technique, the linguistic approach [23] expresses the qualitative information in form of linguistic values of linguistic labels. Let *S* = {*s*_0_, *s*_1_,…, *s*_*g*_} be a linguistic term set. The label *s*_*α*_ represents a possible value of linguistic labels. For instance, a linguistic term set of seven labels could be given as follows:

(1) S=s0=noneN,s1=very  badVB,s2=badB,s3=mediumM,s4=goodG,s5=very  goodVG,s6=perfectP

where the central label *s*_3_ represents the mediocre comment and the others sit on either side of the central one symmetrically and uniformly. Generally, *S* should meet the following features:

1. A negation operator: Neg(*s*_*i*_) = *s*_*g*+1−*i*_.
2. An order: *s*_*i*_ ≥ *s*_*j*_ if and only if *i* ≥ *j*.
3. A max operator: max(*s*_*i*_, *s*_*j*_) = *s*_*i*_ if *s*_*i*_ ≥ *s*_*j*_; a min operator: min(*s*_*i*_, *s*_*j*_) = *s*_*i*_ if *s*_*i*_ ≤ *s*_*j*_.

In order to avoid information loss effectively, Herrera [37] introduced the 2-tuple fuzzy linguistic representation model which is composed of a linguistic label *s*_*i*_ and a real number *α* ∈ [−0.5,0.5) denoting the value of symbolic translation.

**Definition 1 (see [37]).**

Let *S* = {*s*_0_, *s*_1_,…, *s*_*g*_} be a linguistic term set and let *β* ∈ [0, g] be a number value representing the aggregation result of linguistic symbolic. Then the function Δ is defined as follows:

(2) Δ:0,g⟶S×−0.5,0.5

(3) Δβ=si,αwith  siα=β−ii=roundβα∈−0.5,0.5

where round (·) is the integer operator, *s*_*i*_ is the closest index label to *β*, and *α* is the value of symbolic translation.

**Definition 2 (see [37]).**

Let *S* = {*s*_0_, *s*_1_,…, *s*_*g*_} be a linguistic term set and let (*s*_*i*_, *α*_*i*_) be a linguistic 2-tuple. Then the equivalent numerical value *β* ∈ [0, *g*] to a 2-tuple (*s*_*i*_, *α*_*i*_) can be obtained by the following function Δ^−1^ : *S* × [−0.5,0.5)→[0, *g*]

(4) $$\begin{array}{c} \Delta^{-1}\left(\;s_{i},\alpha \;\right)=\alpha +i=\beta \end{array}$$

We can convert a linguistic term to a linguistic 2-tuple by adding a value 0 as symbolic translation:

(5) $$\begin{array}{c} \Delta \left(\;s_{i}\;\right)=\left(\;s_{i},0\;\right). \end{array}$$

The computational model of 2-tuple linguistic information has been developed, such as 2-tuple comparison operator, 2-tuple negation operator, and a wide range of 2-tuple aggregation operators.

### 2.2. The Unbalanced Linguistic Representation Model

The unbalanced linguistic representation model was introduced by Herrera [42]. The advantage of this model is that it can manage the linguistic assessment variables which are nonuniformly and nonsymmetrically distributed.

**Definition 3 (see [42]).**

If a linguistic term set S has a maximum linguistic term, a minimum linguistic term, and a central linguistic term and other terms are nonuniformly and nonsymmetrically distributed around the central one on both left and right lateral sets, i.e., the different discrimination levels on both sides of central linguistic term, then this type of linguistic term sets is called unbalanced linguistic term sets. An unbalanced linguistic term set S can be noted as *S* = *S*_*L*_ ∪ *S*_*C*_ ∪ *S*_*R*_, in which *S*_*C*_ contains the central linguistic term merely and *S*_*L*_ contains all left linguistic terms lower than the central one. Similarly, *S*_*R*_ contains all right linguistic terms higher than the central one.

**Example 4 .**

**Figure 1:**
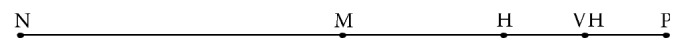
Unbalanced linguistic term set.

When experts try to evaluate the “comfort” of a car, the linguistic assessment set is S= {N (none), M (middle), H (high), VH (very high), P (perfect)}, in which *S*_*L*_ = {*N*}, *S*_*C*_ = {*M*}, *S*_*R*_ = {*H*, *VH*, *P*}. Obviously, it has the minimum linguistic term N, the maximum linguistic term P, and the central linguistic term M, and the number of terms in the left is 1 which is lower than that in the right (3). In other words, discrimination levels on both sides of central linguistic term are different. So S is an unbalanced linguistic term set (Figure 1).

In order to transmit the unbalanced linguistic terms into linguistic 2-tuple information, the concept of a linguistic hierarchy was defined as follows.

**Definition 5 (see [47, 48]).**

A linguistic hierarchy is a set of all levels with each level being a linguistic term set of different granularity. It can be noted as *LH* = ⋃_*t*_*l*(*t*, *n*(*t*)), where *l*(*t*, *n*(*t*)) is a level belonging to the linguistic hierarchy, *t* is a number that indicates the level of the hierarchy, and *n*(*t*) is the granularity of the linguistic term set of t. The set *FP*_*t*_ = {0, 1/2(*n*(*t*) − 1), ⋯, (2*n*(*t*) − 1)/2(*n*(*t*) − 1), 1} is called former modal points set of the level t. The construction of a LH must satisfy linguistic hierarchy basic rules:

  Rule 1: to preserve all former modal points of the membership functions of each linguistic term from one level to the following one.
  Rule 2: to make smooth transitions between successive levels. The aim is to add a new linguistic term set in the hierarchy such that a new linguistic term will be added between each pair of terms belonging to the term set of the previous level t.

**Example 6 .**

**Figure 2:**
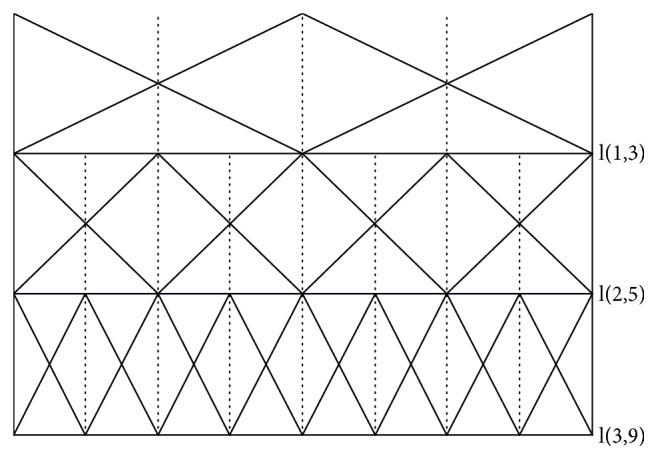
Linguistic hierarchies of 3, 5, and 9 labels.

A linguistic hierarchy of level 3 could be given as follows:

*LH* = *l*(1, 3) ∪ *l*(2, 5) ∪ *l*(3, 9) = {*s*_0_^3^, *s*_1_^3^, *s*_2_^3^}∪{*s*_0_^5^, ⋯, *s*_4_^5^}∪{*s*_0_^9^, ⋯, *s*_8_^9^}, where n(1)=3, n(2)=5, n(3)=9; that is, the first level is a linguistic term set of granularity 3, the second level is a linguistic term set of granularity 5, and the third level is a linguistic term set of granularity 9. It can be graphically shown in Figure 2 with the granularity for each linguistic term set of a LH according to the rules in Table 1.

**Definition 7 (see [49]).**

Let *s*_*i*_^*n*(*t*)^ be a linguistic term of the level *t*, then the transformation function from a linguistic level *t* to another level *t*′ is defined as follows:

(6) TFt′tsint,αnt=Δt′Δt−1sint,αnt·nt′−1nt−1

**Example 8 .**

Let (*s*_2_^5^, 0.3) be a linguistic 2-tuple representation of level 2, and its linguistic 2-tuple representation of level 3 is

(7) TF32s35,0.3=Δ3Δ2−1s25,0.3·9−15−1=Δ34.6=s59,−0.4.

For each label of unbalanced linguistic term set, the semantic representation can be obtained by using linguistic hierarchies. The transformation process is illustrated by the following example.

**Example 9 .**

For an unbalanced linguistic term set S = {N, M, H, VH, P}, it can be transformed to 2-tuple representation according to the following steps.

*Step 1. *Due to *S*_*L*_ = {*N*}, assume #(*S*_*L*_) represents the number of linguistic terms in *S*_*L*_, #(*S*_*L*_) = 1.  ∃*n*(1) = 3 such that (*n*(1) − 1)/2 = 1 = #(*S*_*L*_), and *S*_*L*_ can be represented by a label of level 1 in LH, i.e., *N* = *s*_0_^3^.

*Step 2. *Due to *S*_*R*_ = {*H*, *VH*, *P*}, suppose #(*S*_*R*_) is the number of linguistic labels in *S*_*R*_, #(*S*_*R*_) = 3. ∃*n*(2) = 5, *n*(3) = 9 with (*n*(2) − 1)/2 = 2 < #(*S*_*R*_) < (*n*(3) − 1)/2 = 4, and the semantic representation of *S*_*R*_ can be got from labels of level 3, 4 in LH. Assume *lab*_*i*_ is the number of assigned labels of level i in LH; according to proposition 3 in [42], *lab*_2_ = (*n*(3) − 1)/2 − #(*S*_*R*_) = 1 and *lab*_3_ = 2 can be obtained; that is, *S*_*R*_ can be represented by three labels of level 3 and two labels of level 4; i.e., *H* = *s*_3_^5^, *VH* = *s*_7_^9^, *P* = *s*_8_^9^.

*Step 3. *To bridge representation gaps defined in [42], *S*_*C*_ can be represented by the upside of the central label in level 1 and the downside of level 3, respectively; i.e., $M=\bar{s_{1}^{3}}\cup \underline{s_{4}^{9}}$.

*Step 4. *The ultimate representations are as follows:

(8) SL:N=s03;SC:M=s13¯∪s49_;SR:H=s35,VH=s79,P=s89.

**Definition 10 (see [42]).**

The transformation function from an unbalanced linguistic 2-tuple *s*_*i*_ ∈ *S* to its corresponding linguistic 2-tuple representation *s*_*k*_^*n*(*t*)^ in LH is a mapping

 
*LH* : *S* × [−0.5,0.5 → *LH* × [−0.5,0.5), such that ∀*s*_*i*_ ∈ *S*,  ∃*s*_*k*_^*n*(*t*)^  *LH*(*s*_*i*_, *α*_*i*_) = (*s*_*k*_^*n*(*t*)^, *α*_*i*_).

Conversely, we can obtain the linguistic 2-tuple representation from the unbalanced linguistic 2-tuple:

 
*LH*^−1^ : *LH* × [−0.5,0.5) → *S* × [−0.5,0.5), ∀*s*_*k*_^*n*(*t*)^ ∈ *S*^*n*(*t*)^, *LH*^−1^(*s*_*k*_^*n*(*t*)^, *α*_*i*_) = (*s*_*i*_, *λ*), *λ* can be determined by cases as follows:

(1) If *s*_*i*_(*s*_*i*_ ∈ *S*) is represented by merely one label in LH, then *LH*^−1^(*s*_*k*_^*n*(*t*)^, *α*_*i*_) = (*s*_*i*_, *α*_*i*_),  *λ* = *α*_*i*_.

(2) If *s*_*i*_(*s*_*i*_ ∈ *S*) is represented by two labels in LH, then *λ* = *α*_*i*_ or

(9) λ=Δt−1sknt,αi·nt+1−1nt−1−roundΔt−1sknt,αi·nt+1−1nt−1

(3) If there exists no *s*_*i*_ ∈ *S* such that *s*_*i*_ = *s*_*k*_^*n*(*t*)^, we convert *s*_*k*_^*n*(*t*)^ to another level; that is, *LH*^−1^(*s*_*k*_^*n*(*t*)^, *α*_*i*_) = *LH*^−1^(*TF*_*t*′_^*t*^(*s*_*k*_^*n*(*t*)^, *α*_*i*_)), then return to (1) or (2).

**Example 11 .**

Continuing Example 9, we have

(10) LHP,0=s1617,0,LH−1s59,−0.3=AG,−0.3,LH−1s49,0.2=LH−1TF13s49,0.2=LH−1s13,0.4=M,0.4.

## 3. Some Operational Laws for Unbalanced Linguistic 2-Tuple

Based on 2-tuple representation model, we propose some operational laws and properties of unbalanced linguistic 2-tuple.

**Definition 12 .**

Let (*s*_*i*_, *α*_*i*_) and (*s*_*j*_, *α*_*j*_) be two unbalanced linguistic 2-tuples, *λ* > 0, then one has

1. (*s*_*i*_, *α*_*i*_) ⊕ (*s*_*j*_, *α*_*j*_) = *LH*^−1^(Δ(Δ^−1^(*TF*_*t*_*H*__^*t*_*i*_^(*LH*(*s*_*i*_, *α*_*i*_))) + Δ^−1^(*TF*_*t*_*H*__^*t*_*j*_^(*LH*(*s*_*j*_, *α*_*j*_)))));
2. (*s*_*i*_, *α*_*i*_) ⊗ (*s*_*j*_, *α*_*j*_) = *LH*^−1^(Δ(Δ^−1^(*TF*_*t*_*H*__^*t*_*i*_^(*LH*(*s*_*i*_, *α*_*i*_))) · Δ^−1^(*TF*_*t*_*H*__^*t*_*j*_^(*LH*(*s*_*j*_, *α*_*j*_)))));
3. *λ* · (*s*_*i*_, *α*_*i*_) = *LH*^−1^(Δ(*λ* · Δ^−1^(*TF*_*t*_*H*__^*t*_*i*_^(*LH*(*s*_*i*_, *α*_*i*_)))));
4. (*s*_*i*_, *α*_*i*_)^*λ*^ = *LH*^−1^(Δ(Δ^−1^(*TF*_*t*_*H*__^*t*_*i*_^(*LH*(*s*_*i*_, *α*_*i*_))))^*λ*^).

**Theorem 13 .**

Assume that (*s*_*i*_, *α*_*i*_) and (*s*_*j*_, *α*_*j*_) are two unbalanced linguistic 2-tuples, *λ* > 0, then

1. (*s*_*i*_, 0) ⊕ (*s*_*j*_, 0) = (*s*_*j*_, 0) ⊕ (*s*_*i*_, 0);
2. (*s*_*i*_, 0) ⊗ (*s*_*j*_, 0) = (*s*_*j*_, 0) ⊗ (*s*_*i*_, 0);
3. *λ* · ((*s*_*i*_, 0) ⊕ (*s*_*j*_, 0)) = (*λ*⊙(*s*_*i*_, 0)) ⊕ (*λ*⊙(*s*_*j*_, 0));
4. (*λ*_1_ + *λ*_2_) · (*s*_*i*_, 0) = (*λ*_1_ · (*s*_*i*_, 0)) ⊕ (*λ*_2_ · (*s*_*i*_, 0));
5. ((*s*_*i*_, 0) ⊗ (*s*_*j*_, 0))^*λ*^ = (*s*_*i*_, 0)^*λ*^ ⊗ (*s*_*j*_, 0)^*λ*^;
6. *λ*_1_ · *λ*_2_ · (*s*_*i*_, 0) = (*λ*_1_*λ*_2_) · (*s*_*i*_, 0);
7. (*s*_*i*_, 0)^*λ*_1_^ ⊗ (*s*_*i*_, 0)^*λ*_2_^ = (*s*_*i*_, 0)^*λ*_1_+*λ*_2_^;
8. ((*s*_*i*_, 0)^*λ*_1_^)^*λ*_2_^ = (*s*_*i*_, 0)^*λ*_1_·*λ*_2_^.

**Proof**

(1)

(11) si,0⊕sj,0=LH−1ΔΔ−1TFtHtiLHsi,0+Δ−1TFtHtjLHsj,0=LH−1ΔΔ−1TFtHtjLHsj,0+Δ−1TFtHtiLHsi,0=sj,0⊕si,0;

(2)

(12) si,0⊗sj,0=LH−1ΔΔ−1TFtHtiLHsi,0·Δ−1TFtHtjLHsj,0=LH−1ΔΔ−1TFtHtjLHsj,0·Δ−1TFtHtiLHsi,0=sj,0⊗si,0;

(3)

(13) λ⊙si,0⊕sj,0=LH−1Δλ·Δ−1TFtHtiLHsi,0+Δ−1TFtHtjLHsj,0=LH−1Δλ·Δ−1TFtHtiLHsi,0+λ·Δ−1TFtHtjLHsj,0=λ⊙si,0⊕λ⊙sj,0;

(4)

(14) λ1+λ2⊙si,0=LH−1Δλ1+λ2·Δ−1TFtHtiLHsi,0=LH−1Δλ1·Δ−1TFtHtiLHsi,0+λ2·Δ−1TFtHtiLHsi,0=λ1⊙si,0⊕λ2⊙si,0

(5)

(15) si,0⊗sj,0λ=LH−1ΔΔ−1TFtHtiLHsi,0·Δ−1TFtHtjLHsj,0λ=LH−1ΔΔ−1TFtHtiLHsi,0λ·Δ−1TFtHtjLHsj,0λ=si,0λ⊗sj,0λ;

(6)

(16) λ1⊙λ2⊙si,0=LH−1Δλ1·λ2·Δ−1TFtHtiLHsi,0=λ1λ2⊙si,0;

(7)

(17) si,0λ1⊗si,0λ2=LH−1ΔΔ−1TFtHtiLHsi,0λ1·Δ−1TFtHtiLHsi,0λ2=LH−1ΔΔ−1TFtHtiLHsi,0λ1+λ2=si,0λ1+λ2;

(8)

(18) si,0λ1λ2=LH−1ΔΔ−1TFtHtiLHsi,0λ1λ2=LH−1ΔΔ−1TFtHtiLHsi,αiλ1·λ2=si,0λ1·λ2.

## 4. Some Heronian Mean Operators

### 4.1. The Existing Heronian Mean Operators

The Heronian mean operator has the capacity of capturing the interaction between the input arguments.

**Definition 14 (see [15]).**

Let (*x*_1_, ⋯, *x*_*n*_) be a collection of nonnegative numbers; the Heronian mean operator is a mapping HM: (0, +*∞*)^*n*^ → (0, +*∞*) which satisfies

(19) $$\begin{array}{c} HM\left(\;x_{1},x_{2},\cdots x_{n}\;\right)=\frac{2}{n\left(n+1\right)}\overset{n}{\underset{i=1}{\sum }}\;\overset{n}{\underset{j=i}{\sum }}\sqrt{x_{i}x_{j}} \end{array}$$

A series of HM operators are provided, such as the generalized HM (GHM) operator and the generalized geometric HM (GGHM) operator.

**Definition 15 (see [15]).**

Let (*x*_1_, ⋯, *x*_*n*_) be a collection of nonnegative numbers and *p* ≥ 0, *q* ≥ 0, *p* + *q* > 0; the generalized Heronian mean operator is a mapping GHM: (0, +*∞*)^*n*^ → (0, +*∞*) which satisfies

(20) GHMp,qx1,x2,⋯xn=2nn+1∑i=1n ∑j=inxipxjq1/p+q

**Definition 16 (see [17]).**

Let (*x*_1_, ⋯, *x*_*n*_) be a collection of nonnegative numbers and *p* ≥ 0, *q* ≥ 0, *p* + *q* > 0; the generalized geometric Heronian mean operator is a mapping GGHM: (0, +*∞*)^*n*^ → (0, +*∞*) which satisfies

(21) GGHMp,qx1,x2,⋯xn=1p+q∏i=1n ∏j=inpxi+qxj2/nn+1

### 4.2. The Proposed Heronian Mean Operators

The Heronian mean operator can capture the relevance of individual argument. However, it is rarely applied in unbalanced linguistic information. In this section, we shall extend the Heronian mean operator to the situation in which the input arguments are unbalanced linguistic information and shall develop some unbalanced linguistic Heronian mean operators, such as the unbalanced linguistic generalized arithmetic Heronian mean (ULGAHM) operator, the unbalanced linguistic generalized geometry Heronian mean (ULGGHM) operator, the unbalanced linguistic generalized weighted Heronian mean (ULGWHM) operator, and the unbalanced linguistic generalized weighted geometric Heronian mean (ULGWGHM) operator. Moreover, some properties of these operators are investigated; some special cases with respect to the parameter values are discussed simultaneously.

**Definition 17 .**

Let *p*, *q* ≥ 0, *p* + *q* > 0 and (*s*_*i*_, 0)(*i* = 1, ⋯, *n*) be a collection of unbalanced linguistic 2-tuple variables, then the unbalanced linguistic generalized arithmetic Heronian mean operator of dimension n is a mapping ULGAHM: *Ω*^*n*^ → *Ω*, which satisfies

(22) ULGAHMp,qs1,0,⋯,sn,0=LH−1Δ2nn+1∑i=1n ∑j=inΔ−1TFtHtiLHsi,0pΔ−1TFtHtjLHsj,0q1/p+q

where *Ω* is the set of all unbalanced linguistic 2-tuple variables and *t*_*H*_ is the level of the maximum granularity in LH.

Now, we explore some properties of the ULGAHM operator.

**Theorem 18 .**

Let ((*s*_1_, 0), ⋯, (*s*_*n*_, 0)) be a collection of unbalanced linguistic 2-tuples and *p*, *q* ≥ 0, then the properties of the ULGAHM operator are given as follows:

(1) Monotonicity: let ((*s*_1_, 0), ⋯, (*s*_*n*_, 0)) and ((*s*_1_′, 0), ⋯, (*s*_*n*_′, 0)) be two collections of unbalanced linguistic 2-tuples and (*s*_*i*_, 0) ≥ (*s*_*i*_′, 0) for all *i* = 1, ⋯, *n*, then
  (23) ULGAHMp,qs1,0,⋯,sn,0≥ULGAHMp,qs1′,0,⋯,sn′,0.
(2) Idempotency: if (*s*_*i*_, 0) = (*s*, 0) for all *i* = 1, ⋯, *n*, then
  (24) ULGAHMp,qs1,0,⋯,sn,0=ULGAHMp,qs,0,⋯,s,0=s,0.
(3) Boundedness: ULGAHM operator lies between maximum and minimum operator; i.e.,
  (25) min⁡s1,0,⋯,sn,0≤ULGAHMp,qs1,0,⋯,sn,0≤max⁡s1,0,⋯,sn,0.

**Proof**

(1) Since (*s*_*i*_, 0) ≥ (*s*_*i*_′, 0) for all *i* = 1, ⋯, *n*, according to the definition of LH and Δ,

we have *TF*_*t*_*H*__^*t*_*i*_^(*LH*(*s*_*i*_, 0)) ≥ *TF*_*t*_*H*__^*t*_*i*′  _^(*LH*(*s*_*i*_′, 0)) for all *i* = 1, ⋯, *n*; based on Definition 12,

(26) 2nn+1∑i=1n ∑j=inΔ−1TFtHtiLHsi,0p·Δ−1TFtHtjLHsj,0q1/p+q≥2nn+1∑i=1n ∑j=inΔ−1TFtHti′LHsi′,0p·Δ−1TFtHtj′LHsj′,0q1/p+q

Thus, *ULGAHM*^*p*,*q*^((*s*_1_, 0), ⋯, (*s*_*n*_, 0)) ≥ *ULGAHM*^*p*,*q*^((*s*_1_′, 0), ⋯, (*s*_*n*_′, 0)).

(2) Since (*s*_*i*_, 0) = (*s*, 0) for all *i* = 1, ⋯, *n*, we have

(27) ULGAHMp,qs1,0,⋯,sn,0=LH−1Δ2nn+1∑i=1n ∑j=inΔ−1TFtHtiLHs,0pΔ−1TFtHtiLHs,0q1/p+q=LH−1ΔΔ−1TFtHtiLHs,0=s,0.

(3) Let (*s*_*∗*_, 0) = min⁡((*s*_1_, 0), ⋯, (*s*_*n*_, 0)), (*s*^*∗*^, 0) = max⁡((*s*_1_, 0), ⋯, (*s*_*n*_, 0)); according to the property of idempotency, we have *ULGAHM*^*p*,*q*^((*s*_*∗*_, 0), ⋯, (*s*_*∗*_, 0)) = (*s*_*∗*_, 0), *ULGAHM*^*p*,*q*^((*s*^*∗*^, 0), ⋯, (*s*^*∗*^, 0)) = (*s*^*∗*^, 0), since (*s*_*∗*_, 0) ≤ (*s*_*i*_, 0) ≤ (*s*^*∗*^, 0) for all *i* = 1, ⋯, *n*.

Thus, (*s*_*∗*_, 0) = *ULGAHM*^*p*,*q*^((*s*_*∗*_, 0), ⋯, (*s*_*∗*_, 0)) ≤ *ULGAHM*^*p*,*q*^((*s*_1_, 0), ⋯, (*s*_*n*_, 0)) ≤ *ULGAHM*^*p*,*q*^((*s*^*∗*^, 0), ⋯, (*s*^*∗*^, 0)) = (*s*^*∗*^, 0).

It is easy to see that the unbalanced linguistic generalized arithmetic Heronian mean operator does not satisfy the property of commutativity.

We can get a series of special cases by assigning different values to the parameters *p* and *q* of the ULGAHM operator.

(1) If *q* → 0, we get
  (28) ULGAHMp,0s1,0,⋯,sn,0=LH−1Δ2nn+1∑i=1n ∑j=inΔ−1TFtHtiLHsi,0pΔ−1TFtHtjLHsj,0q1/p+q=LH−1Δ2nn+1∑i=1n ∑j=inΔ−1TFtHtiLHsi,0p1/p=LH−1Δ∑i=1n2n−i+1nn+1Δ−1TFtHtiLHsi,0p1/p=⨁i=1n2n−i+1nn+1⊙si,0p1/p,
which is called the unbalanced linguistic generalized weighted mean (ULGWM) operator with the descending weight vector (2/(*n* + 1), ⋯,2/*n*(*n* + 1))^*T*^.
(2) If *p* = 1, *q* → 0, we have
  (29) ULGAHM1,0s1,0,⋯,sn,0=LH−1Δ2nn+1∑i=1n ∑j=inΔ−1TFtHtiLHsi,0Δ−1TFtHtjLHsj,0q1/q=LH−1Δ2nn+1∑i=1n ∑j=inΔ−1TFtHtiLHsi,0=LH−1Δ∑i=1n2n−i+1nn+1Δ−1TFtHtiLHsi,0=⨁i=1n2n−i+1nn+1⊙si,0.
The ULGAHM operator reduces to the unbalanced linguistic weighted mean (ULWM) operator.
(3) If *p* = 2, *q* → 0, we obtain
  (30) ULGAHM2,0s1,0,⋯,sn,0=LH−1Δ2nn+1∑i=1n ∑j=1nΔ−1TFtHtiLHsi,02Δ−1TFtHtjLHsj,0q1/2+q=LH−1Δ2nn+1∑i=1n ∑j=inΔ−1TFtHtiLHsi,021/2=LH−1Δ∑i=1n2n−i+1nn+1Δ−1TFtHtiLHsi,021/2=⨁i=1n2n−i+1nn+1⊙si,021/2,
which is called the unbalanced linguistic weighted square mean (ULWSM) operator.
(4) If *p* → 0, we have
  (31) ULGAHM0,qs1,0,⋯,sn,0=LH−1Δ2nn+1∑i=1n ∑j=inΔ−1TFtHtiLHsi,0pΔ−1TFtHtjLHsj,0q1/p+q=LH−1Δ2nn+1∑i=1n ∑j=inΔ−1TFtHtjLHsj,0q1/q=LH−1Δ2nn+1∑j=1njΔ−1TFtHtjLHsj,0q1/q=⨁j=1n2jnn+1·sj,0q1/q.
Obviously, the ULGAHM operator reduces to the unbalanced linguistic generalized weighted mean (ULGWM) operator with the ascending weight vector (2/*n*(*n* + 1), ⋯,2/(*n* + 1))^*T*^.
(5) If *p* = *q* = 1, we obtain
  (32) ULGAHM1,1s1,0,⋯,sn,0=LH−1Δ2nn+1∑i=1n ∑j=inΔ−1TFtHtiLHsi,0pΔ−1TFtHtjLHsj,0q1/p+q=LH−1Δ2nn+1∑i=1n ∑j=inΔ−1TFtHtiLHsi,0Δ−1TFtHtjLHsj,01/2=⨁i=1n ⨁j=in2nn+1⊙si,0⊗sj,01/2.
(6) If *p* = *q* = 1/2, we get
  (33) ULGAHM1/2,1/2s1,0,⋯,sn,0=LH−1Δ2nn+1∑i=1n ∑j=inΔ−1TFtHtiLHsi,0pΔ−1TFtHtjLHsj,0q1/p+q=LH−1Δ2nn+1∑i=1n ∑j=inΔ−1TFtHtiLHsi,01/2Δ−1TFtHtjLHsj,01/2=⨁i=1n ⨁j=in2nn+1⊙si,01/2⊗sj,01/2,
which we call general unbalanced linguistic Heronian mean (ULHM) operator in this case.

We introduce the concept of the unbalanced linguistic generalized geometric Heronian mean operator as follows.

**Definition 19 .**

Let *p*, *q* ≥ 0,  *p* + *q* > 0, and (*s*_*i*_, 0)(*i* = 1, ⋯, *n*) be a collection of unbalanced linguistic 2-tuples, then the unbalanced linguistic generalized geometric Heronian mean operator of dimension n is a mapping ULGGHM:  *Ω*^*n*^ → *Ω*, which satisfies

(34) ULGGHMp,qs1,0,⋯,sn,0=LH−1Δ1p+q∏i=1n ∏j=inpΔ−1TFtHtiLHsi,0+qΔ−1TFtHtjLHsj,02/nn+1

where *Ω* is the set of all the unbalanced linguistic 2-tuples and *t*_*H*_ is a level of the maximum granularity in LH.

Some properties of the unbalanced linguistic generalized geometric Heronian mean operator are investigated as follows.

**Theorem 20 .**

Let ((*s*_1_, 0), ⋯, (*s*_*n*_, 0)) be a collection of unbalanced linguistic 2-tuples and *p*, *q* ≥ 0, then the properties of the ULGGHM operator are given as follows:

(1) Monotonicity: let ((*s*_1_, 0), ⋯, (*s*_*n*_, 0)) and ((*s*_1_′, 0), ⋯, (*s*_*n*_′, 0)) be two collections of unbalanced 2-tuple linguistic variables and (*s*_*i*_, 0) ≥ (*s*_*i*_′, 0) for all *i* = 1, ⋯, *n*, then
  (35) ULGGHMp,qs1,0,⋯,sn,0≥ULGGHMp,qs1′,0,⋯,sn′,0.
(2) Idempotency: if (*s*_*i*_, 0) = (*s*, 0) for all *i* = 1, ⋯, *n*, then
  (36) ULGGHMp,qs1,0,⋯,sn,0=ULGGHMp,qs,0,⋯,s,0=s,0.
(3) Boundedness: let (*s*_*∗*_, 0) = min⁡((*s*_1_, 0), ⋯, (*s*_*n*_, 0)), (*s*^*∗*^, 0) = max⁡((*s*_1_, 0), ⋯, (*s*_*n*_, 0)), then (*s*_*∗*_, 0) ≤ *ULGGHM*^*p*,*q*^((*s*_1_, 0), ⋯, (*s*_*n*_, 0)) ≤ (*s*^*∗*^, 0).

**Proof**

The proof of Theorem 20 can be seen in the Appendix.

Similarly, the unbalanced linguistic generalized geometric Heronian mean operator does not satisfy the property of commutativity.

Next, we analyze some particular cases in regard to parameters *p* and *q*.

(1) If *q* → 0, then
  (37) ULGGHMp,0s1,0,⋯,sn,0=LH−1Δ1p+q∏i=1n ∏j=inpΔ−1TFtHtiLHsi,0+qΔ−1TFtHtjLHsj,02/nn+1=LH−1Δ∏j=1nΔ−1TFtHtiLHsi,02n+i−1/nn+1=⨂i=1nsi,02n−i+1/nn+1,
which we call the unbalanced linguistic geometric mean (ULGM) operator with the descending weight vector. It has no relationship with p while *q* → 0.
(2) If *p* → 0, then
  (38) ULGGHM0,qs1,0,⋯,sn,0=LH−1Δ1p+q∏i=1n ∏j=inpΔ−1TFtHtiLHsi,0+qΔ−1TFtHtjLHsj,02/nn+1=LH−1Δ∏j=1nΔ−1TFtHtjLHsj,02j/nn+1=⨂j=1nsj,02j/nn+1.
The ULGGHM operator reduces to the unbalanced linguistic geometric mean (ULGM) operator with the ascending weight vector. It has no relationship with q while *p* → 0.
(3) If *p* = *q* = 1, then
  (39) ULGGHM1,1s1,0,⋯,sn,0=LH−1Δ1p+q∏i=1n ∏j=inpΔ−1TFtHtiLHsi,0+qΔ−1TFtHtjLHsj,02/nn+1=LH−1Δ12∏i=1n ∏j=inΔ−1TFtHtiLHsi,0+Δ−1TFtHtjLHsj,02/nn+1=⨂i=1n⨂j=in12⊙si,0⊕sj,02/nn+1.
(4) If *p* = *q* = 1/2, then
  (40) ULGGHM1/2,1/2s1,0,⋯,sn,0=LH−1Δ1p+q∏i=1n ∏j=inpΔ−1TFtHtiLHsi,0+qΔ−1TFtHtjLHsj,02/nn+1=LH−1Δ∏i=1n ∏j=in12Δ−1TFtHtiLHsi,0+12Δ−1TFtHtjLHsj,02/nn+1=⨂i=1n⨂j=in12⊙si,0⊕12⊙sj,02/nn+1,
which we call general unbalanced linguistic geometric Heronian mean (ULHM) operator in this case.

In (22) and (34), all aggregated arguments have the same importance. However, different parameters have different importance because of the different attitudes of decision-makers. Considering the importance of each argument, so we introduce the unbalanced linguistic generalized weighted arithmetic Heronian mean (ULGWAHM) operator and the unbalanced linguistic generalized weighted geometric Heronian mean (ULGWGHM) operator as follows.

**Definition 21 .**

Let *p*, *q* ≥ 0, *p* + *q* > 0, and (*s*_*i*_, 0)(*i* = 1, ⋯, *n*) be a collection of unbalanced linguistic 2-tuples, then the unbalanced linguistic generalized weighted arithmetic Heronian mean operator of dimension n is a mapping ULGWAHM: *Ω*^*n*^ → *Ω*, which satisfies

(41) ULGWAHMp,qs1,0,⋯,sn,0=LH−1Δ∑i=1n∑j=inwiΔ−1TFtHtiLHsi,0pwjΔ−1TFtHtjLHsj,0q1/p+q∑i=1n∑j=inwipwjq1/p+q

where *Ω* is the set of all the unbalanced linguistic 2-tuples, *t*_*H*_ is a level of the maximum granularity in LH, and *W* = (*w*_1_, ,⋯,*w*_*n*_)^*T*^ is the weight vector of (*s*_*i*_, 0)(*i* = 1, ⋯, *n*), satisfying *w*_*i*_ ≥ 0, ∑_*i*=1_^*n*^*w*_*i*_ = 1.

Now, we discuss some properties of the unbalanced linguistic generalized weighted arithmetic Heronian mean operator.

**Theorem 22 .**

Assume that ((*s*_1_, 0), ⋯, (*s*_*n*_, 0)) is a collection of unbalanced linguistic 2-tuples and *p*, *q* ≥ 0, then the properties of the unbalanced linguistic generalized weighted arithmetic Heronian mean operator are given as follows:

(1) Reducibility: let *w*_1_ = ⋯ = *w*_*n*_ = 1/*n*, then
  (42) ULGWAHMp,qs1,0,⋯,sn,0=ULGAHMp,qs1,0,⋯,sn,0.
(2) Monotonicity: let ((*s*_1_, 0), ⋯, (*s*_*n*_, 0)) and ((*s*_1_′, 0), ⋯, (*s*_*n*_′, 0)) be two collections of unbalanced linguistic 2-tuples and (*s*_*i*_, 0) ≥ (*s*_*i*_′, 0) for all *i* = 1, ⋯, *n*, then
  (43) ULGWAHMp,qs1,0,⋯,sn,0≥ULGWAHMp,qs1′,0,⋯,sn′,0.
(3) Idempotency: if (*s*_*i*_, 0) = (*s*, 0) for all *i* = 1, ⋯, *n*, then
  (44) ULGWAHMp,qs1,0,⋯,sn,0=ULGWAHMp,qs,0,⋯,s,0=s,0.
(4) Boundedness: let (*s*_*∗*_, 0) = min⁡((*s*_1_, 0), ⋯, (*s*_*n*_, 0)), (*s*^*∗*^, 0) = max⁡((*s*_1_, 0), ⋯, (*s*_*n*_, 0)), then
  (45) s∗,0≤ULGWAHMp,qs1,0,⋯,sn,0≤s∗,0.

It is easy to see that the unbalanced linguistic generalized weighted arithmetic Heronian mean operator does not satisfy the property of commutativity.

**Proof**

The proof of Theorem 22 and some special cases of the unbalanced linguistic generalized weighted arithmetic Heronian mean operator in regard to parameters *p* and *q* can be seen in the Appendix.

Considering the importance of input arguments and unbalanced linguistic generalized geometric Heronian mean operator, we further introduce the unbalanced linguistic generalized weighted geometric Heronian mean (ULGWGHM) operator.

**Definition 23 .**

Let *p*, *q* ≥ 0,  *p* + *q* > 0, and (*s*_*i*_, 0)(*i* = 1, ⋯, *n*) be a collection of unbalanced linguistic 2-tuples, then the unbalanced linguistic generalized weighted geometric Heronian mean operator of dimension n is a mapping ULGWGHM: *Ω*^*n*^ → *Ω*, which satisfies

(46) ULGWGHMp,qs1,0,⋯,sn,0=LH−1Δ1p+q∏i=1n ∏j=inp·Δ−1TFtHtisi,0+q·Δ−1TFtHtjsj,02n−i+1/nn+1·wj/∑k=inwk

where *Ω* is the set of all the unbalanced linguistic 2-tuples, *t*_*H*_ is a level of LH which has the maximum granularity, and *W* = (*w*_1_, ,⋯,*w*_*n*_)^*T*^ is the weight vector of (*s*_*i*_, 0)(*i* = 1, ⋯, *n*) satisfying *w*_*i*_ ≥ 0, ∑_*i*=1_^*n*^*w*_*i*_ = 1.

Some properties of the unbalanced linguistic generalized weighted geometric Heronian mean operator are investigated as follows.

**Theorem 24 .**

Suppose that ((*s*_1_, 0), ⋯, (*s*_*n*_, 0)) is a collection of unbalanced linguistic 2-tuples and *p*, *q* ≥ 0, then the properties of the ULGWGHM operator are given as follows:

(1) Reducibility: let *w*_1_ = ⋯ = *w*_*n*_ = 1/*n*, then
  (47) ULGWGHMp,qs1,0,⋯,sn,0=ULGGHMp,qs1,0,⋯,sn,0.
(2) Monotonicity: let ((*s*_1_, 0), ⋯, (*s*_*n*_, 0)) and ((*s*_1_′, 0), ⋯, (*s*_*n*_′, 0)) be two collections of unbalanced linguistic 2-tuples and (*s*_*i*_, 0) ≥ (*s*_*i*_′, 0) for all *i* = 1, ⋯, *n*, then
  (48) ULGWGHMp,qs1,0,⋯,sn,0≥ULGWGHMp,qs1′,0,⋯,sn′,0.
(3) Idempotency: if (*s*_*i*_, 0) = (*s*, 0) for all *i* = 1, ⋯, *n*, then
  (49) ULGWGHMp,qs1,0,⋯,sn,0=ULGWGHMp,qs,0,⋯,s,0=s,0.
(4) Boundedness: let (*s*_*∗*_, 0) = min⁡((*s*_1_, 0), ⋯, (*s*_*n*_, 0)), (*s*^*∗*^, 0) = max⁡((*s*_1_, 0), ⋯, (*s*_*n*_, 0)), then
  (50) s∗,0≤ULGWGHMp,qs1,0,⋯,sn,0≤s∗,0.

**Proof**

The proof of Theorem 24 is similar to that of Theorem 22, so it is omitted.

Similarly, the unbalanced linguistic generalized weighted geometric Heronian mean operator does not satisfy the property of commutativity. Some special cases of the unbalanced linguistic generalized weighted geometric Heronian mean operator in regard to parameters *p* and *q* can be seen in the Appendix.

## 5. MAGDM Method Based on Unbalanced Linguistic Information

In this section, we will develop the process of solving MADM problem by using the new proposed unbalanced linguistic Heronian mean operators and entropy measure method to solve MADM problems, where the weights of attributes are unknown, and the assessment values are unbalanced linguistic terms.

### 5.1. Problem Description

A MADM problem can be described as a quadruple 〈*X*, *E*, *D*, *S*〉, where

*X* = {*x*_1_, ⋯, *x*_*m*_} is a set of all possible alternatives for decision-makers and *m* ≥ 2;

*E* = {*e*_1_, ⋯, *e*_*n*_} is a set of attributes for each alternative, and the attributes are supposed to be correlative in this paper;

*D* = {*d*_1_, ⋯, *d*_*t*_} is a set of decision-makers;

*S* ^(*k*)^ = (*s*_*ij*_^(*k*)^)_*m*×*n*_ is the decision matrix provided by the kth decision-maker *d*_*k*_, and *s*_*ij*_^(*k*)^ represents the preference value of *x*_*i*_ with respect to attributes *e*_*j*_, *s*_*ij*_^(*k*)^taking the form of unbalanced linguistic terms.

### 5.2. Entropy Method to Determine the Attribute Weights

An important step of the MADM problem is the determination of the attribute weights. We first introduce the concept of entropy for unbalanced linguistic term sets, then an optimization model is constructed to determine the weights of attributes.

**Definition 25 .**

Let *S* = {*s*_1_, ⋯, *s*_*g*_} be an unbalanced linguistic term set, *X* = {*x*_1_, ⋯, *x*_*n*_} be a finite set, and a mapping *L*_*U*_ : *X* → *S*, then a pair (*X*, *L*_*U*_) is called unbalanced linguistic fuzzy set, and the value *L*_*U*_(*x*) is said to be the grade of unbalanced linguistic membership of *x* in (*X*, *L*_*U*_).

In particular, if *L*_*U*_(*x*_*i*_) is the maximum label or the minimum label, i.e., *L*_*C*_ = {*L*_*U*_(*x*_*i*_) = *s*_1_  or  *s*_*g*_, *x*_*i*_ ∈ *X*, *i* = 1, ⋯, *n*}, then the unbalanced linguistic fuzzy set will reduce to a crisp set.

If all unbalanced linguistic fuzzy sets are denoted as *L*_*U*_*FS*_*S*_(*X*), then the concept of entropy for unbalanced linguistic information can be developed as follows.

**Definition 26 .**

Let *X* = {*x*_1_, ⋯, *x*_*n*_} be a finite set, *L*_*U*_1__ = {*L*(*x*_*i*_) = *s*_*α*_*i*__, *x*_*i*_ ∈ *X*} and *L*_*U*_2__ = {*L*(*x*_*i*_) = *s*_*β*_*i*__, *x*_*i*_ ∈ *X*} be two unbalanced linguistic term sets defined in X, and $\bar{L_{U}\left(x\right)}$ be the negation of *L*_*U*_(*x*); *E*(*L*_*U*_(*x*)) is said to be an entropy measure for unbalanced linguistic term set if the following properties are valid:

1. 0 ≤ *E*(*L*_*U*_(*x*)) ≤ 1;
2. *E*(*L*_*U*_(*x*)) = 0 if and only if *L*_*U*_(*x*) is a crisp set in X, i.e., *L*_*U*_(*x*) = {*L*_*U*_(*x*_*i*_) = *s*_1_  or  *s*_*g*_, *x*_*i*_ ∈ *X*};
3. *E*(*L*_*U*_(*x*)) is a unique maximum if *s*_*i*_ is the central label *s*_*c*_;
4. *L*_*U*_1__ ≥ *L*_*U*_2__ if *s*_*β*_*i*__ ≥ *s*_*α*_*i*__ for *s*_*α*_*i*__ ≥ *s*_*c*_ or if *s*_*β*_*i*__ ≤ *s*_*α*_*i*__ for *s*_*α*_*i*__ ≤ *s*_*c*_;
5. $E\left(\bar{L_{U}\left(x\right)}\right)=E\left(L_{U}\left(x\right)\right)$.

According to Definition 26, we can construct the following formula as the entropy measure of the unbalanced linguistic information:

(51) ELUx=41n∑i=1nΔ−1TFtHtiLHsi,0gtH·1−Δ−1TFtHtiLHsi,0gtH

The entropy of unbalanced linguistic information under each attribute could be calculated according to (51), which is denoted as *E*(*L*_*U*_(*x*_*j*_)), *j* = 1, ⋯, *n*.

The ideal of entropy method is that if the entropy among all attributes for an alternative is larger, then the effective information is less; thus the attributes should be assigned less weight; otherwise, it should be assigned a greater weight.

If the information about weight *w*_*j*_ of the attribute *e*_*j*_(*j* = 1, ⋯, *n*) is completely unknown, we can build the following equations to determine the attribute weight:

(52) $$\begin{array}{c} w_{j}=\frac{1-E\left(L_{U}\left(x_{j}\right)\right)}{\sum_{j=1}^{n}\left(1-E\left(L_{U}\left(x_{j}\right)\right)\right)},\;j=1,\cdots ,n \end{array}$$

Sometimes, the decision-makers only offer partly known information about attribute weights; let Φ be the set of the partly known information about attribute weights. In order to get an objective attribute weight vector, the optimal model based on maximum entropy principle could be constructed:

(53) max Ew=λ∑j=1nwjELUxj+1−λ−∑j=1nwjln⁡wjs.t. w1,⋯,wn∈Φ 0≤wj≤1,

where *λ* ∈ [0,1] is an attribute parameter representing the attitude of the decision-makers toward one of the objectives.

### 5.3. The Decision-Making Procedure

To obtain the best opinion, the new approach based on the new unbalanced linguistic Heronian mean operator is presented to solve the MADM problem. The new approach involved the following steps.

**Step 1 .**

Calculate the semantic representation about the unbalanced linguistic variables.

The semantic representation can be calculated by

(54) $$\begin{array}{c} LH:S\times \left[\;-0.5,0.5\;\right)⟶LH\times \left[\;-0.5,0.5\;\right). \end{array}$$

**Step 2 .**

Translate the unbalanced linguistic variable *s*_*ij*_ to a linguistic 2-tuple variable.

A linguistic 2-tuple variable can be obtained as (*r*_*ij*_, *α*_*ij*_), where *r*_*ij*_ = Δ(*round*(Δ^−1^(*LH*(*s*_*ij*_)))),  *α*_*ij*_ = Δ^−1^(*LH*(*s*_*ij*_)) − *round*(Δ^−1^(*LH*(*s*_*ij*_))).

**Step 3 .**

Calculate the entropy of unbalanced linguistic values under all attributes.

The entropy of unbalanced linguistic values under ith attribute can be calculated by

(55) ELUxj=41n∑j=1nΔ−1TFtHtiLHsj,0gtH·1−Δ−1TFtHtiLHsj,0gtH.

**Step 4 .**

Generate the attribute weight vector *W* = {*w*_1_, ⋯, *w*_*n*_}.

Once the linguistic 2-tuple variables and entropy are obtained, the attribute weights can be generated by the optimal model:

(56) max Ew=λ∑j=1nwj1−ELUxj+1−λ−∑j=1nwjln⁡wjs.t. w1,⋯,wn∈Φ 0≤wj≤1.

**Step 5 .**

Output the comprehensive assessment values for each alternative.

Based on the weights obtained in Step 4, utilize the ULGWAHM operator or ULGWGHM operator to obtain the comprehensive evaluation values in terms of unbalanced linguistic term for each alternative

(57) $$\begin{array}{c} \left(\;\gamma_{i},\alpha_{i}\;\right)=ULGWAHM\left(\;\left(\;\gamma_{i1},\alpha_{i1}\;\right),\cdots ,\left(\;\gamma_{in},\alpha_{in}\;\right)\;\right); \end{array}$$

or

(58) $$\begin{array}{c} \left(\;\gamma_{i},\alpha_{i}\;\right)=ULGWGHM\left(\;\left(\;\gamma_{i1},\alpha_{i1}\;\right),\cdots ,\left(\;\gamma_{in},\alpha_{in}\;\right)\;\right). \end{array}$$

**Step 6 .**

Rank all alternatives.

Compare the overall values of all alternatives according to unbalanced linguistic comparison laws and select the best one.

**Step 7 .**

End.

## 6. Illustrative Example

In this section, we employ an unbalanced linguistic selection model of low-carbon tourism (LCTD) (adapted from [50]) by applying the proposed MADM method and give an example to demonstrate its effectiveness and validity.

### 6.1. Background

Over the past several decades, economic development has been recognized as the only approach to improve quality of life and social status in communities and cities of different areas, especially in developing countries. Along with rapid social and economic development, problems of carbon emissions are getting serious and have been of critical concern to both national and local governments worldwide for many decades. Increasing numbers of carbon emissions issues (such as global warming, air pollution, sea levels rising, glaciers melting, and Nino phenomenon) can lead to a variety of impacts on and liabilities in public health and sustainable regional development. As a significant part of economic development, the tourism industry is encouraging low-carbon tourism and developing low-carbon tourism destinations (LCTDs). Low-carbon tourism is a “green” form of tourism that is based on the goals of low-energy consumption, low pollution, and low emissions. Therefore, it is important for tourists to select the best option(s) from multiple low-carbon tourism destinations based on multiple attributes while considering carbon reduction, lower energy consumption, and environmental protection because of their ability to protect environment and public health.

Next, we would like to employ an illustrative example to provide certain reference attributes for unbalanced linguistic selection of LCTDs by applying the proposed MAGDM approach.

### 6.2. Case Study

#### 6.2.1. The Establishment of Assessment Systems for LCTDs

In order to mitigate the damage of carbon emissions and save energy, many low-carbon tourism destinations have been developed. Moreover, many tourists have recognized the importance of low-carbon tourism for environmental protection. In order to find a good balance between the enjoyment of a trip and carbon emission reduction, it is crucial for tourists to compare and evaluate some known low-carbon tourism destinations and then choose the best one(s) from these options. Generally speaking, this evaluation and selection process is based on several criteria or attributes. In this case study, the attributes consist of the following four aspects:

 
*e*_1_: low-carbon transportation, low-energy consumption vehicles, and pick-up and drop-off services as reflected in connecting different scenic sites and reaching the destination.
 
*e*_2_: hotels and accommodation, as reflected in green-material labels, low-carbon facilities, and a low-carbon environment and education management. Food service including green food, a low-carbon environment, and low-energy waste handling mechanisms.
 
*e*_3_: consumption satisfaction, as reflected in the service cost of travel agencies, ticket prices for scenic sites, and the cost of accommodation.
 
*e*_4_: attraction and impact of scenic sites, including low-carbon customer service and low-carbon management and control.

In order to select the best LCTD, a tourist (i.e., decision-makers) wants to go on a low-carbon trip. After preliminary screening, there are four low-carbon tourism destinations as the set of alternatives. Therefore, in this case study, the tourist is empowered to provide preferences in terms of several unbalanced linguistic terms on the response alternatives *x*_*i*_(*i* = 1, ⋯, 4) under the four attributes *e*_*j*_(*j* = 1, ⋯, 4). Assume that the four alternatives are to be evaluated using the following unbalanced linguistic term set* S*= {*N (none), L (low), M (medium), AH (almost high), H (high), QH (quite high), VH (very high), AT (almost total), T (total)*}, the density is extreme, *t*_*H*_ = 4, and a linguistic hierarchy is *LH* = *l*(1, 3) ∪ *l*(2, 5) ∪ *l*(3, 9) ∪ *l*(4,17) = {*s*_0_^3^, *s*_1_^3^, *s*_2_^3^} ∪ {*s*_0_^5^, ⋯, *s*_4_^5^}∪{*s*_0_^9^, ⋯, *s*_8_^9^}∪{*s*_0_^17^, ⋯, *s*_16_^17^}.

To obtain the most preferred LCTD, we present the effective MADM approach for the problem, where attribute weights are partly unknown due to the problem complexity. Then the performance unbalanced linguistic assessments for each alternative *x*_*i*_(*i* = 1, ⋯, 4) are listed in Table 2. According to the approach developed in Section 5 and the given parameters, we can rank the order of alternatives by applying the MATLAB software package. The concrete steps are shown as follows.

#### 6.2.2. Procedure of MAGDM Problem Based on Unbalanced Linguistic Heronian Mean Operators

We adopt the proposed method to rank the alternatives in the example and select the best one. The decision steps are as follows.

**Step 1 .**

Calculate the semantic representations which are in form of unbalanced linguistic terms and they are shown as

(59) N=s05,L=s15,M=s25,AH=s59,H=s69,QH=s1317,VH=s1417,AT=s1517,T=s1617.

**Step 2 .**

Translate the unbalanced linguistic variable *s*_*ij*_^(*k*)^ to the linguistic 2-tuple variables which are expressed as

(60) N,0=s05,0,L,0=s15,0,M,0=s25,0,AH,0=s59,0,H,0=s69,0,QH,0=s1317,0,VH,0=s1417,0,AT,0=s1517,0,T,0=s1617,0.

**Step 3 .**

Calculate the entropy of unbalanced linguistic values under each attribute.

Utilizing (52), the entropy of each attribute can be derived as follows:

(61) E1LUxj=0.906,E2LUxj=0.797E3LUxj=0.809,E4LUxj=0.914.

**Step 4 .**

Generate the attribute weight vector.

Utilizing the optimal model (53) and the Lingo 11.0 software package, the attribute weights can be derived with *λ* = 1/2 as follows:

(62) w1=0.2648,w2=0.2793,w3=0.1943,w4=0.2615.

**Step 5 .**

Output the comprehensive assessment values for each alternative.

Utilizing the ULGWAHM operator with the parameters *p* = *q* = 1, we can get the overall collective preference value (*γ*_*i*_, *α*_*i*_) of the alternative *x*_*i*_

(63) γ1,α1=AH,−0.2941,γ2,α2=AH,−0.16355,γ3,α3=M,−0037775,γ4,α4=AH,−0.2283.

**Step 6 .**

We can get *x*_2_≻*x*_4_≻*x*_1_≻*x*_3_; therefore the alternative *x*_2_ is the best choice.

### 6.3. Sensitivity Analysis of the Parameters in Unbalanced Linguistic Heronian Mean Aggregation Operators

#### 6.3.1. Sensitivity Analysis of the Parameters p or q

**Figure 3:**
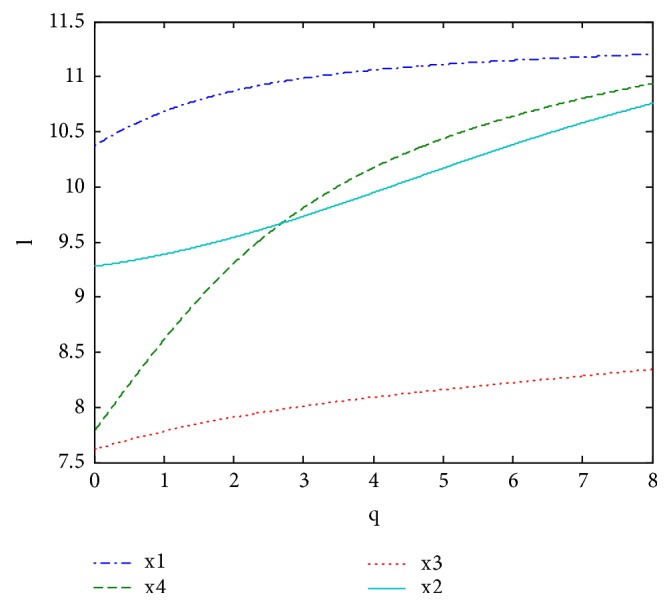
Comprehensive value obtained by ULGWAHM** (***p* = 0, *q* ∈ (0,8]**)**.

**Figure 4:**
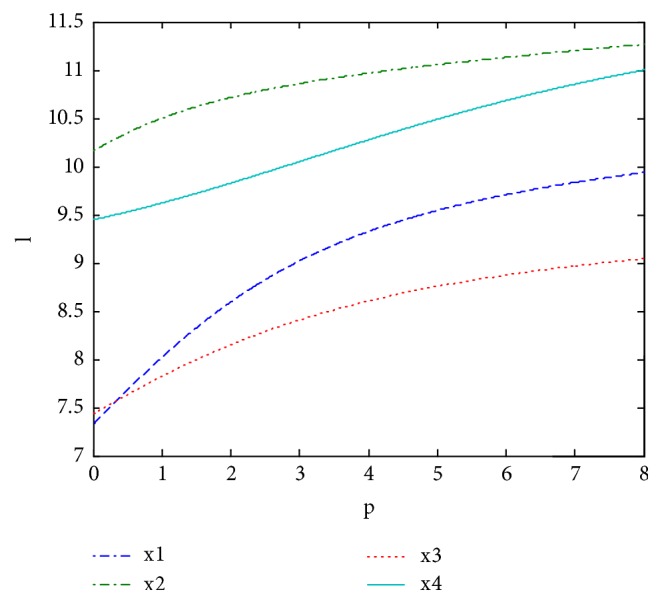
Comprehensive value obtained by ULGWAHM** (***q* = 0, *p* ∈ (0,8]**)**.

In order to illustrate the impact of the parameters p and q on aggregation results, we fixed one of p and q, and different rankings of alternatives and decision-making based on ULGWAHM operator can be obtained which were shown in Figures 3 and 4.

We can find from Figure 3 that

1. when *q* ∈ (0,0.6960], we have *x*_1_≻*x*_2_≻*x*_4_≻*x*_3_ and the best alternative is *x*_1_;
2. when *q* ∈ (2.6960,8], we have *x*_1_≻*x*_4_≻*x*_2_≻*x*_3_ and the best alternative is *x*_1_.
3. We can find from Figure 4 that
4. when *p* ∈ (0,0.35], we have *x*_2_≻*x*_4_≻*x*_3_≻*x*_1_ and the optimal alternative is *x*_2_;
5. when *p* ∈ (0.35,8], we have *x*_2_≻*x*_4_≻*x*_1_≻*x*_3_ and the optimal alternative is *x*_2_.

From Figures 3 and 4, we can see that the larger the value of p or q is, the larger the aggregated value is. Therefore, proper selection can be made according to the attitude of the decision-makers. For instance, in practical decision problems, the decision-maker who is pessimistic can choose the smaller values of the parameters p and q while the optimistic one can choose the bigger values of the parameters p and q.

#### 6.3.2. Sensitivity Analysis of the Parameters p and q

**Figure 5:**
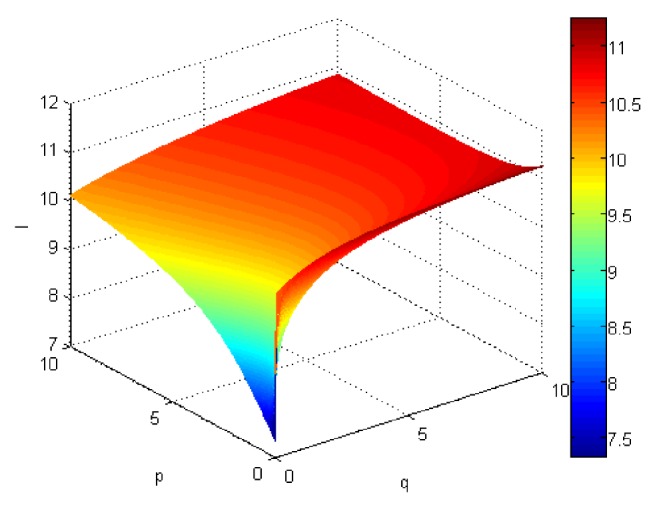
Comprehensive values for alternative x_1_ obtained by ULGWAHM operator (*p* ∈ [0, 10], *q* ∈ [0,10]).

**Figure 6:**
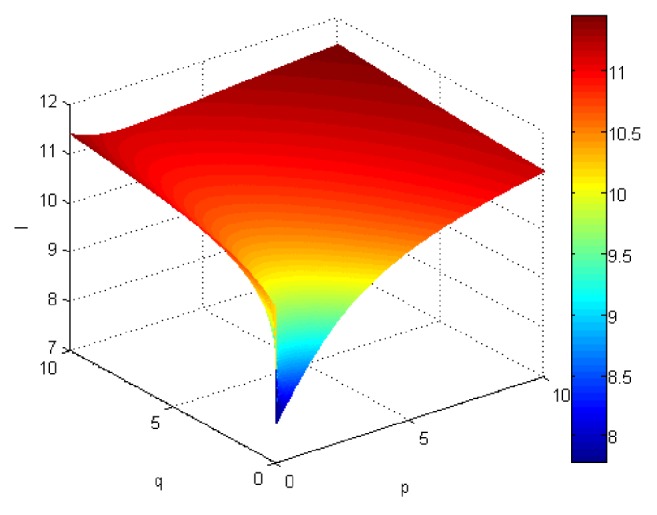
Comprehensive values for alternative x_2_ obtained by ULGWAHM operator (*p* ∈ [0, 10], *q* ∈ [0,10]).

**Figure 7:**
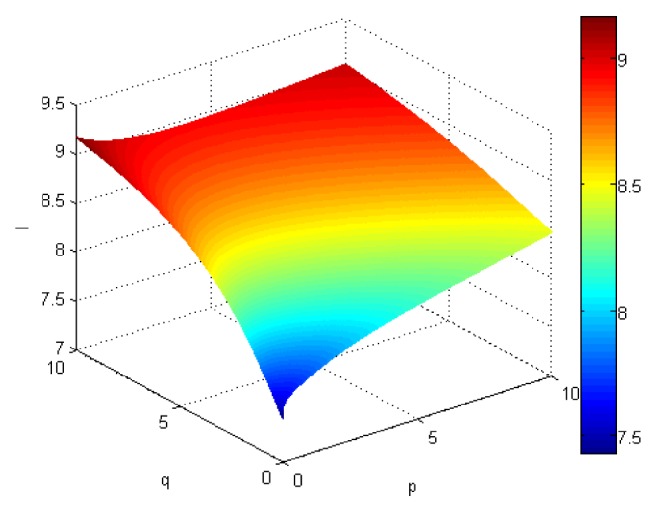
Comprehensive values for alternative x_3_ obtained by ULGWAHM operator (*p* ∈ [0, 10], *q* ∈ [0,10]).

**Figure 8:**
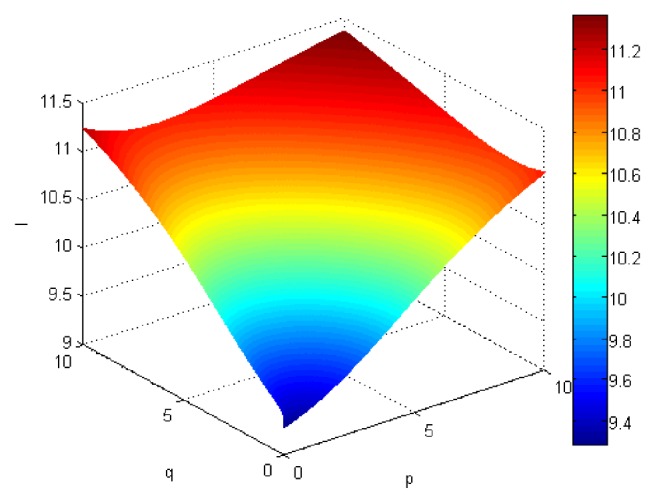
Comprehensive values for alternative x_4_ obtained by ULGWAHM operator (*p* ∈ [0, 10], *q* ∈ [0,10]).

If we let the parameters p and q change simultaneously, the associated aggregation results of each alternative could be obtained which are shown in Figures 5–8.

From Figures 5–8, we can find that the interaction of arguments becomes stronger as values of parameters p and q increase. Therefore, the manager can choose suitable values p and q to determine the optimal alternative based on the practical need and his/her preference.

**Figure 9:**
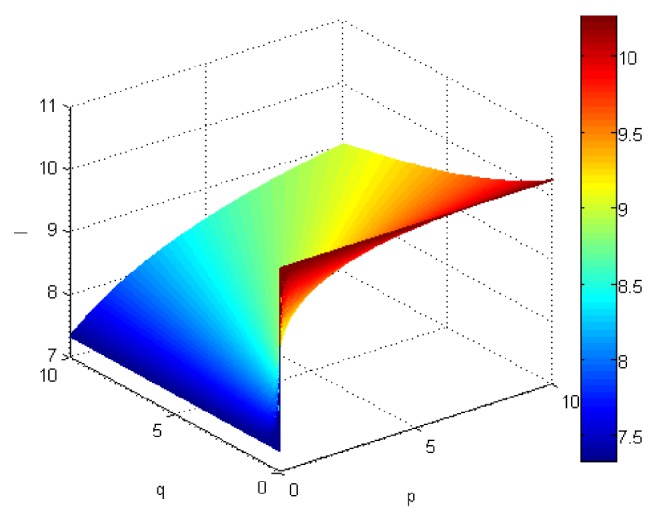
Comprehensive values for alternative x_1_ obtained by ULGWGHM operator (*p* ∈ [0, 10], *q* ∈ [0,10]).

**Figure 10:**
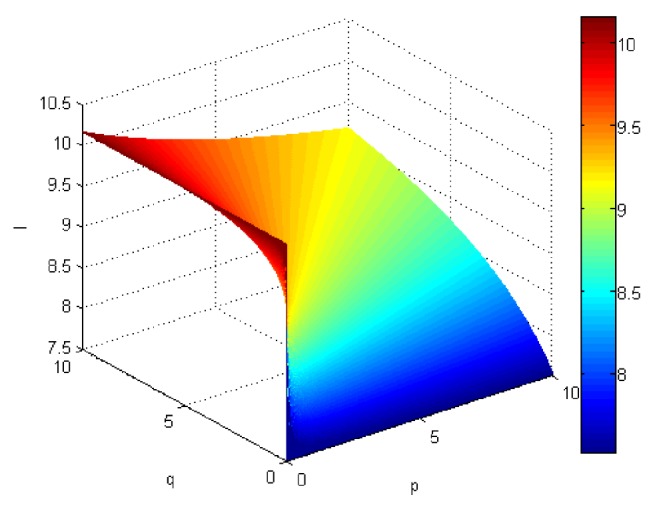
Comprehensive values for alternative x_2_ obtained by ULGWGHM operator (*p* ∈ [0, 10], *q* ∈ [0,10]).

**Figure 11:**
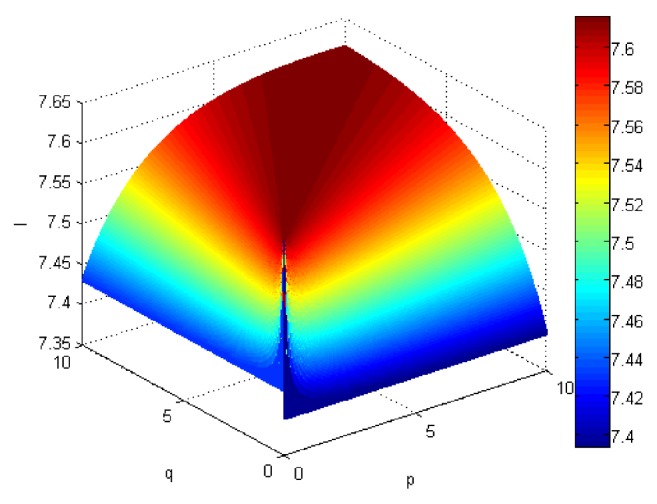
Comprehensive values for alternative x_3_ obtained by ULGWGHM operator (*p* ∈ [0, 10], *q* ∈ [0,10]).

**Figure 12:**
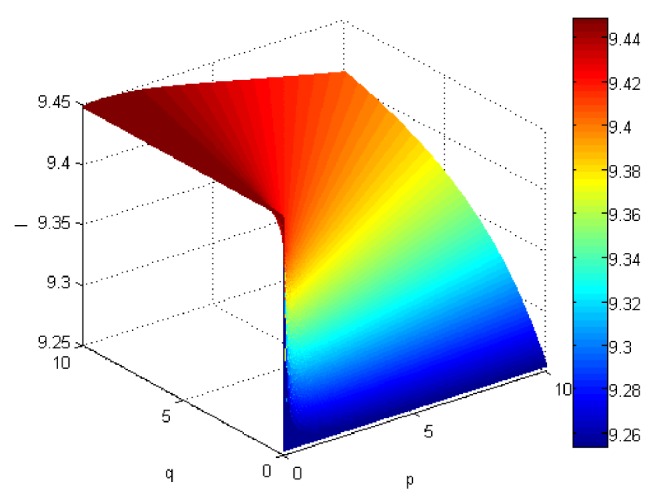
Comprehensive values for alternative x_4_ obtained by ULGWGHM operator (*p* ∈ [0, 10], *q* ∈ [0,10]).

If we use ULGWGHM operator replacing ULGWAHM operator in the above investment, we could obtain the overall collective preference value of each alternative with p=q=1 shown in Table 3 and the aggregation values of each alternative as the values p and q changed simultaneously in Figures 9–12. Obviously, in most cases, the values by ULGWGHM operator are smaller than that of ULGWAHM one for the same aggregation arguments which denote the former is pessimistic while the latter is optimistic. Thus, the decision-maker can suitably select the best alternative according to his/her preference to meet real need. It is of crucial importance in practical decision-making.

From the above analysis, we can choose appropriate value of parameters p, q and the suitable operator to meet the various actual requirements. Consequently, it is more feasible and flexible for decision-making problems.

## 7. Comparison Analyses of the Results Obtained

In this section, a set of comparative studies was conducted with the relevant frequently used aggregation approach and classical decision-making method to demonstrate the feasibility and applicability of the proposed unbalanced linguistic MADM method of this paper.

### 7.1. Comparison with the Existing Linguistic Aggregation Operators

Firstly, we compare our methods with previous 2-tuple linguistic aggregation operators including the dependent 2-tuple ordered weighted average (D2TOWA) operator and the dependent 2-tuple ordered weight geometric (D2TOWG) operator [31], and the 2-tuple weighted Bonferroni mean (2TWBM) operator and the 2-tuple weighted geometric Bonferroni mean (2TWGBM) operator [51].

Wei [31] proposed the concepts of series dependent 2-tuple (D2TL) aggregation operators and a linguistic MADM problem with 2-tuple linguistic information. In order to use the D2TOWA, D2TOWG, 2TWBM, and 2TWGBM operators, the evaluation values of this paper should be transformed into 2-tuple linguistic information which are as follows:

(64) N⟶s05,0,L⟶s15,0,M⟶s25,0,AH⟶s59,0,H⟶s69,0,QH⟶s1317,0,VH⟶s1417,0,AT⟶s1517,0,T⟶s1617,0.

**Figure 13:**
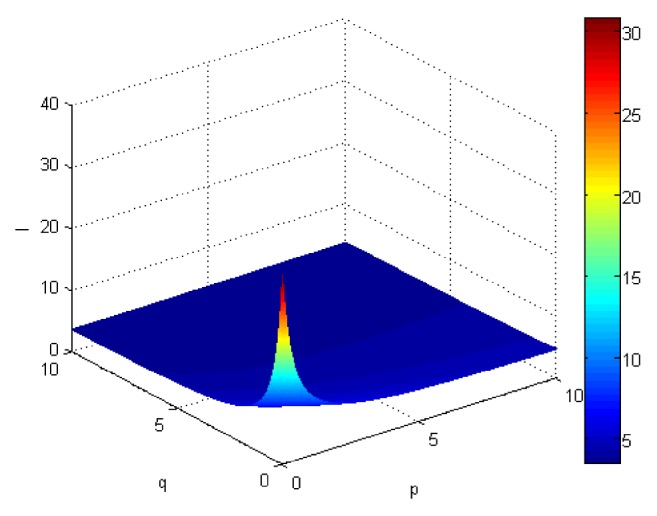
Comprehensive values for alternative x_1_ obtained by ULWBM operator (*p* ∈ [0, 10], *q* ∈ [0,10]).

**Figure 14:**
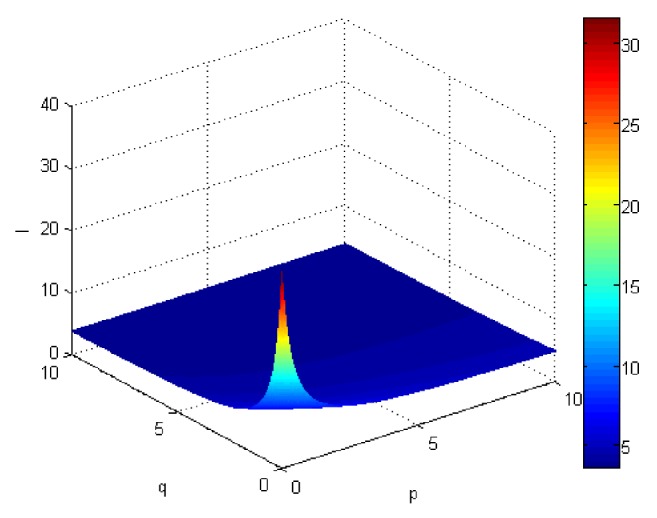
Comprehensive values for alternative x_2_ obtained by ULWBM operator (*p* ∈ [0, 10], *q* ∈ [0,10]).

**Figure 15:**
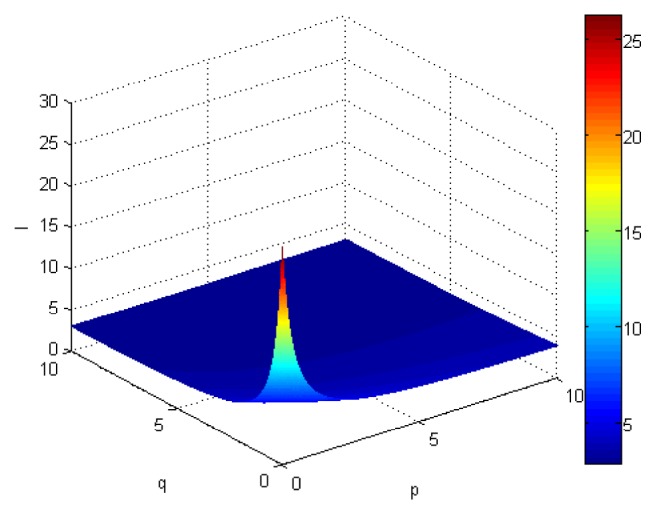
Comprehensive values for alternative x_3_ obtained by ULWBM operator (*p* ∈ [0, 10], *q* ∈ [0,10]).

**Figure 16:**
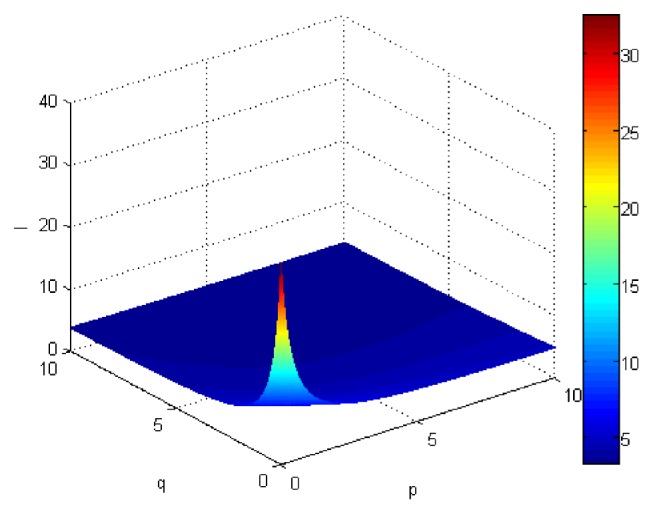
Comprehensive values for alternative x_4_ obtained by ULWBM operator (*p* ∈ [0, 10], *q* ∈ [0,10]).

After calculating the dependent weights *w*_11_ = 0.3182, *w*_12_ = 0.2576, *w*_13_ = 0.2576, *w*_14_ = 0.1667, *w*_21_ = 0.3261, *w*_22_ = 0.2681, *w*_23_ = 0.2391, *w*_24_ = 0.1667, *w*_31_ = 0.3333, *w*_32_ = 0.2500, *w*_33_ = 0.2500, *w*_34_ = 0.1667, *w*_41_ = 0.3056, *w*_42_ = 0.2500, *w*_43_ = 0.2500, *w*_44_ = 0.1944, the overall collective preference can be obtained. The comparison is shown in Table 4 (p=q=1). The aggregation results of four alternatives by ULWBM operator as the parameters *p* and *q* changed simultaneously in Figures 13–16.

Compared with the existing 2-tuple linguistic aggregation operators, our proposed approaches have the following advantages:

(1) The approach in this paper considers the interactions between not only criteria values *e*_*i*_ and *e*_*j*_  (*i* < *j*) but also between *e*_*i*_ and itself, while the method of [31] cannot process and [51] ignores the correlation between *e*_*i*_ and itself. Thus, the method in this paper is more effective than the others.

(2) The proposed method of this paper has flexible parameters p and q. We can choose the appropriate values of parameters to satisfy the real demand. But the method in [31] has no selectable parameters. Thus, the proposed method is more flexible.

(3) The method in [31, 51] can only deal with the case where the input arguments are the form of 2-tuple whereas ours is suitable for three cases: linguistic variables, 2-tuple, and unbalanced linguistic information which indicates that ours is more universal.

### 7.2. The TOPSIS Method for Unbalanced Linguistic MAMD

In the following, we will put emphasis on the classical TOPSIS method. The basic principle of the TOPSIS method is that the optimal alternative should have the farthest distance from the negative ideal solution and the closest distance from the positive ideal solution simultaneously. The steps are involved by using the TOPSIS method.

**Step 1 .**

We can obtain the 2-tuple linguistic representation shown in Section 6.2.2.

**Step 2 .**

Define the unbalanced linguistic positive ideal solution (ULPIS) and the negative ideal solution (ULNIS). Since the unbalanced linguistic term set is* S*= {*N (none), L (low), M (medium), AH (almost high), H (high), QH (quite high), VH (very high), AT (almost total), T (total)*}, thus the ULPIS and ULNIS are *r*^−^ = *N* and *r*^+^ = *T*, respectively.

**Step 3 .**

Calculate the distance from each evaluation value to ULPIS and ULNIS using the following equation:

(65) di+=∑j=1nwjdrij,r+,di−=∑j=1nwjdrij,r−

where the separation between alternatives is the Hamming distance; i.e., *d*(*r*_*ij*_, *r*^−^) = |Δ^−1^(*TF*_*t*_*h*__^*t*^(*LH*(*r*_*ij*_))) − Δ^−1^(*TF*_*t*_*h*__^*t*^(*LH*(*r*^−^)))|, then we can get *d*_*i*_^+^ and *d*_*i*_^−^. It is obvious that the larger *d*_*i*_^−^ and the smaller *d*_*i*_^+^, the better the alternative.

**Step 4 .**

Calculate the closeness coefficient to ideal solution as

(66) $$\begin{array}{c} CC_{i}=\frac{d_{i}^{-}}{d_{i}^{+}+d_{i}^{-}},\;i=1,2,3,4 \end{array}$$

The closeness coefficient to ideal solution for the alternative *x*_*i*_ can be obtained as

(67) CC1=d1+d1++d1−=0.5442,CC2=d2−d2++d2−=0.6469,CC3=d3−d3++d3−=0.4876,CC4=d4−d4++d4−=0.5941

According to the closeness coefficient, we can determine the ranking of all alternatives as *x*_2_≻*x*_4_≻*x*_1_≻*x*_3_, and the best alternative is *x*_2_.

Obviously, the ranking of alternatives obtained by the unbalanced linguistic TOPSIS method is identical to that by the ULGWAHM aggregation operator, which states the validity of the proposed method in this paper. Thus, response solution *x*_2_ is the most appropriate one.

According to the comparison that focuses on different angles, we find the result based on the ULGWAHM operator is identical to other operators and TOPSIS methods. In fact, these methods have their own advantages and disadvantages correspondingly. In summary, the ULGAHM model proposed in this paper has the following characteristics:

(1) The proposed method of this paper is suitable for series linguistic information, including linguistic variables, 2-tuple, and unbalanced linguistic information which is universal.

(2) The approach in this paper considers the correlation of all attributes; simultaneously it has flexible parameters to satisfy the complex decision-making problems. Thus, the proposed method is flexible.

(3) We propose a model to deal with the situation where the weights information is unknown. The proposed model for optimal weight vector is advantaged and effective, which takes objective weights information into consideration.

In summary, the developed method would be more suitable to handle indeterminate information and unbalanced information in complex decision-making problems. Therefore, it is more reasonable than existing methods.

## 8. Conclusions

This paper focuses on MADM problem with unbalanced linguistic information, which introduced some new unbalanced linguistic Heronian mean aggregation functions by using unbalanced linguistic information and Heronian mean operator. First, we have presented the ULGAHM operator and the ULGGHM operator. Then, the ULGWAHM operator and ULGWGHM operator have been proposed in consideration of different importance of attributes. These operators are very helpful in situations where the assessed information can not be expressed with real number but with unbalanced linguistic information. Some main properties and particular cases of the operators have been studied. We have applied the new method for investment projects and made the selection based on the new aggregation operators. It is easy to find that the results are the identical one with the special ULGWAHM operator and ULGWGHM operator.

In the future, we expect to extend unbalanced linguistic Heronian mean operator to other situations, such as interval linguistic information, intuitionistic fuzzy linguistic environment, and more complicated situation, and consider other applications.

## Figures and Tables

**Table 1:** Linguistic hierarchies.

Level	t=1	t=2	t=3
Granularity	n(t)=3	n(t)=5	n(t)=9

**Table 2:** Decision matrix with unbalanced linguistic information.

	*e*_1_	*e*_2_	*e*_3_	*e*_4_
X_1_	L	AH	H	H
X_2_	AH	H	QH	L
X_3_	AH	L	AH	M
X_4_	M	H	AH	M

**Table 3:** The collective overall preference values obtained by the proposed method.

	*x*_1_	*x*_2_	*x*_3_	*x*_4_
ULGWAHM	LH^−1^(Δ(9.4118)) (AH,−0.2941)	LH^−1^(Δ(9.6729)) (AH,−0.16355)	LH^−1^Δ(7.8489)) (M,−0.037775)	LH^−1^(Δ(9.5434) (AH,−0.2283)
Ranking	*x*_2_≻*x*_4_≻*x*_1_≻*x*_3_			
ULGWGHM	LH^−1^(Δ(7.3211)) (M,−0.169725)	LH^−1^(Δ(10.1566)) (AH,0.1566)	LH^−1^Δ(7.4289)) (M,−0.142775)	LH^−1^(Δ(9.4471)) (AH,−0.27645)
Ranking	*x*_2_≻*x*_4_≻*x*_3_≻*x*_1_			

**Table 4:** Comparison with the existing linguistic operators.

Aggregation operators	Linguistic distribution	Parameter number	Order of alternative
D2TOWA operator	Balanced	One	*x*_2_≻*x*_1_≻*x*_4_≻*x*_3_
D2TOWG operator	Balanced	One	*x*_2_≻*x*_1_≻*x*_4_≻*x*_3_
2TWBM operator	Balanced	Two	*x*_2_≻*x*_1_≻*x*_4_≻*x*_3_
2TWGBM operator	Balanced	Two	*x*_2_≻*x*_1_≻*x*_4_≻*x*_3_
ULGWAHM operator	Unbalanced	Two	*x*_2_≻*x*_4_≻*x*_1_≻*x*_3_
ULGWGHM operator	Unbalanced	Two	*x*_2_≻*x*_4_≻*x*_3_≻*x*_1_

## Appendix

### A. The Proof of Theorem 20

Proof (1) Since (*s*_*i*_, 0) ≥ (*s*_*i*_′, 0) for all *i* = 1, ⋯, *n*, then *TF*_*t*_*H*__^*t*_*i*_^(*LH*(*s*_*i*_, 0)) ≥ *TF*_*t*_*H*__^*t*_*i*′_^(*LH*(*s*_*i*_′, 0)), *TF*_*t*_*H*__^*t*_*j*_^(*LH*(*s*_*j*_, 0)) ≥ *TF*_*t*_*H*__^*t*_*j*_′^(*LH*(*s*_*j*_′, 0)) for all *i* = 1, ⋯, *n*,

(A.1) 1p+q∏i=1n ∏j=inpΔ−1TFtHtiLHsi,0+qΔ−1TFtHtjLHsj,02/nn+1≥1p+q∏i=1n ∏j=inpΔ−1TFtHti′LHsi′,0+qΔ−1TFtHtj′LHsj′,02/nn+1

Thus, *ULGGHM*^*p*,*q*^((*s*_1_, 0), ⋯, (*s*_*n*_, 0)) ≥ *ULGGHM*^*p*,*q*^((*s*_1_′, 0), ⋯, (*s*_*n*_′, 0)). (2) Since (*s*_*i*_, 0) = (*s*, 0) for all *i* = 1, ⋯, *n*, then

(A.2) ULGGHMp,qs1,0,⋯,sn,0=LH−1Δ1p+q∏i=1n ∏j=inpΔ−1TFtHtiLHsi,0+qΔ−1TFtHtjLHsj,02/nn+1=LH−1ΔΔ−1TFtHtiLHs,0=s,0.

(3) According to the property of idempotency and monotonicity, we have (*s*_*∗*_, 0) = *ULGGHM*^*p*,*q*^((*s*_*∗*_, 0), ⋯, (*s*_*∗*_, 0)) ≤ *ULGAHM*^*p*,*q*^((*s*_1_, 0), ⋯, (*s*_*n*_, 0)), (*s*^*∗*^, 0) = *ULGAHM*^*p*,*q*^((*s*^*∗*^, 0), ⋯, (*s*^*∗*^, 0)) ≥ *ULGAHM*^*p*,*q*^((*s*_1_, 0), ⋯, (*s*_*n*_, 0)); i.e., (*s*_*∗*_, 0) ≤ *ULGAHM*^*p*,*q*^((*s*_1_, 0), ⋯, (*s*_*n*_, 0)) ≤ (*s*^*∗*^, 0).

### B. The Proof of Theorem 22

Proof (1) Since *w*_1_ = ⋯ = *w*_*n*_ = 1/*n*, according to (41), we have

(B.1) ULGWAHMp,qs1,0,⋯,sn,0=LH−1Δ∑i=1n∑j=in1/n·Δ−1TFtHtiLHsi,0p1/n·Δ−1TFtHtjLHsj,0q1/p+q∑i=1n∑j=in1/np1/nq1/p+q=LH−1Δ1/n∑i=1n∑j=inΔ−1TFtHtiLHsi,0pΔ−1TFtHtjLHsj,0q1/p+q1/n·nn+1/21/p+q=LH−1Δ2nn+1∑i=1n ∑j=inΔ−1TFtHtiLHsi,0pΔ−1TFtHtjLHsj,0q1/p+q=ULGAHMp,qs1,0,⋯,sn,0.

(2) Since (*s*_*i*_, 0) ≥ (*s*_*i*_′, 0) for all *i* = 1, ⋯, *n*, *p*, *q* ≥ 0. Then

(B.2) wi·Δ−1TFtHtiLHsi,0p≥wi·Δ−1TFtHti′LHsi′,0p,wj·Δ−1TFtHtjLHsj,0q≥wj·Δ−1TFtHtj′LHsj′,0q.

Further,

(B.3) ∑i=1n∑j=inwi·Δ−1TFtHtiLHsi,0pwj·Δ−1TFtHtjLHsj,0q1/p+q∑i=1n∑j=inwipwjq1/p+q≥∑i=1n∑j=inwi·Δ−1TFtHti′LHsi′,0pwj·Δ−1TFtHtj′LHsj′,0q1/p+q∑i=1n∑j=inwipwjq1/p+q

Thus, *ULGWAHM*^*p*,*q*^((*s*_1_, 0), ⋯, (*s*_*n*_, 0)) ≥ *ULGWAHM*^*p*,*q*^((*s*_1_′, 0), ⋯, (*s*_*n*_′, 0)). (3) Since (*s*_*i*_, 0) = (*s*, 0) for all *i* = 1, ⋯, *n*, then

(B.4) ULGWAHMp,qs1,0,⋯,sn,0=LH−1Δ∑i=1n∑j=inwi·Δ−1TFtHtLHs,0pwj·Δ−1TFtHtLHs,0q1/p+q∑i=1n∑j=inwipwjq1/p+q=LH−1ΔΔ−1TFtHtiLHs,0=s,0.

(4) According to the property of idempotency and monotonicity, we can get (*s*_*∗*_, 0) ≤ *ULGWAHM*^*p*,*q*^((*s*_1_, 0), ⋯, (*s*_*n*_, 0)) ≤ (*s*^*∗*^, 0).

### C. Some Special Cases of the ULGWAHM Operator in regard to Parameters *p* and *q*

(1) If *q* → 0, then
  (C.1) ULGWAHMp,0s1,0,⋯,sn,0=LH−1Δ∑i=1n∑j=inwi·Δ−1TFtHtiLHsi,0pwj·Δ−1TFtHtjLHsj,0q1/p+q∑i=1n∑j=inwipwjq1/p+q=LH−1Δ∑i=1n∑j=inwi·Δ−1TFtHtiLHsi,0p1/p∑i=1n∑j=inwip1/p=LH−1Δ∑i=1nn+i−1wip∑i=1nn+i−1wipΔ−1TFtHtiLHsi,0p1/p=⨁i=1nn+i−1wip∑i=1nn+i−1wip⊙si,0p1/p,
which is called the unbalanced linguistic generalized weighted mean (ULGWM) operator.
(2) If *p* → 0, then
  (C.2) ULGWAHM0,qs1,0,⋯,sn,0=LH−1Δ∑i=1n∑j=inwi·Δ−1TFtHtiLHsi,0pwj·Δ−1TFtHtjLHsj,0q1/p+q∑i=1n∑j=inwipwjq1/p+q=LH−1Δ∑i=1n∑j=inwj·Δ−1TFtHtjLHsj,0q1/q∑i=1n∑j=inwjq1/q=LH−1Δ∑j=1njwjq∑j=1njwjq·Δ−1TFtHtjLHsj,0q1/q=⨁j=1njwjq∑j=1njwjq⊙sj,0q1/q.
The ULGWAHM reduces to the unbalanced linguistic generalized weighted mean (ULGWM) operator.
(3) If *p* = *q* = 1, then
  (C.3) ULGWAHM1,1s1,0,⋯,sn,0=LH−1Δ∑i=1n∑j=inwi·Δ−1TFtHtiLHsi,0wj·Δ−1TFtHtjLHsj,01/2∑i=1n∑j=inwiwj1/2=⨁i=1n⨁j=inwi∑i=1n∑j=inwiwj⊙si,01/2⊗wj∑i=1n∑j=inwiwj⊙sj,01/2.
(4) If *p* = *q* = 1/2, we obtain
  (C.4) ULGWAHM1/2,1/2s1,0,⋯,sn,0=LH−1Δ∑i=1n∑j=inwi·Δ−1TFtHtiLHsi,01/2wj·Δ−1TFtHtjLHsj,01/2∑i=1n∑j=inwi1/2wj1/2=⨁i=1n⨁j=inwi1/2∑i=1n∑j=inwi1/2wj1/2⊙si,01/2⊗wj1/2∑i=1n∑j=inwi1/2wj1/2⊙sj,01/2,
which we call the unbalanced linguistic general weighted mean (ULGWM) operator.

### D. Some Special Cases of the ULGWGHM Operator in regard to Parameters *p* and *q*

(1) If *q* → 0, then
  (D.1) =LH−1Δ1p∏i=1n ∏j=inp·Δ−1TFtHtisi,02n−i+1/nn+1·wj/∑k=inwk=LH−1Δ∏i=1nΔ−1TFtHtiLHsi,02n+i−1/nn+1=⨂i=1nsi,02n−i+1/nn+1,
which we call the unbalanced linguistic geometric mean (ULGM) operator with the descending weight vector. It has no relationship with p while *q* → 0.
(2) If *p* → 0, then
  (D.2) ULGWGHM0,qs1,0,⋯,sn,0=LH−1Δ1q∏i=1n ∏j=inq·Δ−1TFtHtjsj,02n−i+1/nn+1·wj/∑k=inwk=LH−1Δ∏i=1n ∏j=inΔ−1TFtHtjsj,02n−i+1/nn+1·wj/∑k=inwk=⨂i=1n⨂j=insj,02n−i+1/nn+1·wj/∑k=inwk.
The ULGWGHM operator reduces to the unbalanced linguistic weighted geometric mean (ULWGM) operator. It has no relationship with q while *p* → 0.
(3) If *p* = *q* = 1, then
  (D.3) ULGWGHM1,1s1,0,⋯,sn,0=LH−1Δ12∏i=1n ∏j=inΔ−1TFtHtisi,0+Δ−1TFtHtjsj,02n−i+1/nn+1·wj/∑k=inwk=12⊙⨂i=1n⨂j=insi,0⊕sj,02n−i+1/nn+1·wj/∑k=inwk.
(4) If *p* = *q* = 1/2, then
  (D.4) ULGWGHM1/2,1/2s1,0,⋯,sn,0=LH−1Δ12∏i=1n ∏j=inΔ−1TFtHtisi,0+Δ−1TFtHtjsj,02n−i+1/nn+1·wj/∑k=inwk=12⊙si,0⊕12⊙sj,02n−i+1/nn+1·wj/∑k=inwk,
which we call unbalanced linguistic weighted geometric Heronian mean (ULWGHM) operator in this case.

## References

[1] Guo K., Li W. (2011). Combination rule of D-S evidence theory based on the strategy of cross merging between evidences. *Expert Systems with Applications*.

[2] Yager R. R. (1988). On ordered weighted averaging aggregation operators in multicriteria decisionmaking. *The Institute of Electrical and Electronics Engineers Systems, Man, and Cybernetics Society*.

[3] Xu Z. S., Da Q. L. (2002). The ordered weighted geometric averaging operators. *International Journal of Intelligent Systems*.

[4] Xu Z. (2008). Dependent uncertain ordered weighted aggregation operators. *Information Fusion*.

[5] Xu Z. S., Da Q. L. (2002). The uncertain OWA operator. *International Journal of Intelligent Systems*.

[6] Yager R. R. (2004). Generalized OWA aggregation operators. *Fuzzy Optimization and Decision Making*.

[7] Zhou L.-G., Chen H.-Y. (2011). Continuous generalized OWA operator and its application to decision making. *Fuzzy Sets and Systems*.

[8] Merigó J. M., Casanovas M. (2011). Induced and uncertain heavy OWA operators. *Computers & Industrial Engineering*.

[9] Merigó J. M., Gil-Lafuente A. M. (2009). The induced generalized OWA operator. *Information Sciences*.

[10] Liao H., Xu Z. (2014). Some new hybrid weighted aggregation operators under hesitant fuzzy multi-criteria decision making environment. *Journal of Intelligent & Fuzzy Systems*.

[11] Liao H., Xu Z. (2015). Extended hesitant fuzzy hybrid weighted aggregation operators and their application in decision making. *Soft Computing*.

[12] Liu P., Wang P. (2018). Some q-Rung Orthopair Fuzzy Aggregation Operators and their Applications to Multiple-Attribute Decision Making. *International Journal of Intelligent Systems*.

[13] Beliakov G., Pradera A., Calvo T. (2007). *Aggregation Functions: A Guide for Practitioners*.

[14] Sykora S. (2009). *Mathematical Means and Averages: Generalized Heronian Means*.

[15] Sykora S. (2009). *Generalized Heronian Means II*.

[16] Yu D. J. (2013). Intuitionistic fuzzy geometric Heronian mean aggregation operators. *Applied Soft Computing*.

[17] Liu P., Liu Z., Zhang X. (2014). Some intuitionistic uncertain linguistic Heronian mean operators and their application to group decision making. *Applied Mathematics and Computation*.

[18] Chu Y., Liu P. (2015). Some two-dimensional uncertain linguistic Heronian mean operators and their application in multiple-attribute decision making. *Neural Computing and Applications*.

[19] Liu P., Liu J., Merigó J. M. (2018). Partitioned Heronian means based on linguistic intuitionistic fuzzy numbers for dealing with multi-attribute group decision making. *Applied Soft Computing*.

[20] Liu P., Chen S. M. (2017). Group decision making based on Heronian aggregation operators of intuitionistic fuzzy numbers. *IEEE Transactions on Cybernetics*.

[21] Xu Z. (2015). *Uncertain Multi-Attribute Decision Making: Methods and Applications*.

[22] Xu Z.-S., Chen J. (2007). An interactive method for fuzzy multiple attribute group decision making. *Information Sciences*.

[23] Zadeh L. A. (1975). The concept of a linguistic variable and its application to approximate reasoning I. *Information Sciences*.

[24] Liu P., Chen S.-M. (2018). Multiattribute group decision making based on intuitionistic 2-tuple linguistic information. *Information Sciences*.

[25] Liu P. (2017). Multiple attribute group decision making method based on interval-valued intuitionistic fuzzy power Heronian aggregation operators. *Computers & Industrial Engineering*.

[26] Liu P. D., Liu J., Chen S. M. (2018). Some intuitionistic fuzzy Dombi Bonferroni mean operators and their application to multi-attribute group decision making. *Journal of the Operational Research Society*.

[27] Liu P., Chen S.-M., Liu J. (2017). Multiple attribute group decision making based on intuitionistic fuzzy interaction partitioned Bonferroni mean operators. *Information Sciences*.

[28] Liu P., Li H. (2017). Interval-valued intuitionistic fuzzy power bonferroni aggregation operators and their application to group decision making. *Cognitive Computation*.

[29] Merigó J. M., Casanovas M., Martínez L. (2010). Linguistic aggregation operators for linguistic decision making based on the Dempster-Shafer theory of evidence. *International Journal of Uncertainty, Fuzziness and Knowledge-Based Systems*.

[30] Pei Z., Ruan D., Liu J., Xu Y. (2012). A linguistic aggregation operator with three kinds of weights for nuclear safeguards evaluation. *Knowledge-Based Systems*.

[31] Wei G., Zhao X. (2012). Some dependent aggregation operators with 2-tuple linguistic information and their application to multiple attribute group decision making. *Expert Systems with Applications*.

[32] Wan S.-P. (2013). 2-Tuple linguistic hybrid arithmetic aggregation operators and application to multi-attribute group decision making. *Knowledge-Based Systems*.

[33] Wan S.-P. (2013). Some hybrid geometric aggregation operators with 2-tuple linguistic information and their applications to multi-attribute group decision making. *International Journal of Computational Intelligence Systems*.

[34] Xu Z. (2004). A method based on linguistic aggregation operators for group decision making with linguistic preference relations. *Information Sciences*.

[35] Pang Q., Wang H., Xu Z. (2016). Probabilistic linguistic term sets in multi-attribute group decision making. *Information Sciences*.

[36] Wang J. Q., Li J. J. (2009). The multi-criteria group decision making method based on multi-granularity intuitionistic two semantics. *Science and Technology Information*.

[37] Herrera F., Martínez L. (2000). A 2-tuple fuzzy linguistic representation model for computing with words. *IEEE Transactions on Fuzzy Systems*.

[38] Liao H., Xu Z., Zeng X.-J. (2014). Distance and similarity measures for hesitant fuzzy linguistic term sets and their application in multi-criteria decision making. *Information Sciences*.

[39] Xu Z., Wang H. (2017). On the syntax and semantics of virtual linguistic terms for information fusion in decision making. *Information Fusion*.

[40] Rodriguez R. M., Martinez L., Herrera F. (2012). Hesitant fuzzy linguistic term sets for decision making. *IEEE Transactions on Fuzzy Systems*.

[41] Herrera-Viedma E., López-Herrera A. G. (2007). A model of an information retrieval system with unbalanced fuzzy linguistic information. *International Journal of Intelligent Systems*.

[42] Herrera F., Herrera-Viedma E., Martínez L. (2008). A fuzzy linguistic methodology to deal with unbalanced linguistic term sets. *IEEE Transactions on Fuzzy Systems*.

[43] Zou L., Pei Z., Karimi H. R., Shi P. (2012). The unbalanced linguistic aggregation operator in group decision making. *Mathematical Problems in Engineering*.

[44] Meng D., Pei Z. (2013). On weighted unbalanced linguistic aggregation operators in group decision making. *Information Sciences*.

[45] Jiang L., Liu H., Cai J. (2015). The power average operator for unbalanced linguistic term sets. *Information Fusion*.

[46] Pei Z., Shi P. (2011). Fuzzy risk analysis based on linguistic aggregation, operators. *International Journal of Innovative Computing, Information and Control*.

[47] Cordón O., Herrera F., Zwir I. (2002). Linguistic modeling by hierarchical systems of linguistic rules. *IEEE Transactions on Fuzzy Systems*.

[48] Martínez L., Rodriguez R. M., Herrera F. (2015). *A 2-Tuple Linguistic Model Computing with Words in Decision Making*.

[49] Herrera F., Martínez L. (2001). A model based on linguistic 2-tuples for dealing with multigranular hierarchical linguistic contexts in multi-expert decision-making. *IEEE Transactions on Systems, Man, and Cybernetics, Part B: Cybernetics*.

[50] Hui L., Zhou J. W. (2017). Linguistic multi-Attribute group decision making with risk preferences and its use in low-carbon tourism destination selection. *International Journal of Environment Research and Publication Health*.

[51] Liu J. P., Lin S., Chen H. Y. (2013). 2-tuple linguistic Bonferroni aggregation operators and their applications to multi-attribute group decision making. *Operations research and Management Science*.

